# Identification of novel metabolic interactions controlling carbon flux from xylose to ethanol in natural and recombinant yeasts

**DOI:** 10.1186/s13068-015-0340-x

**Published:** 2015-09-25

**Authors:** Gert Trausinger, Christoph Gruber, Stefan Krahulec, Christoph Magnes, Bernd Nidetzky, Mario Klimacek

**Affiliations:** Institute of Biotechnology and Biochemical Engineering, Graz University of Technology, NAWI Graz, Petersgasse 12/1, 8010 Graz, Austria; HEALTH-Institute for Biomedicine and Health Sciences, Joanneum Research Forschungsgesellschaft m.b.H., Graz, Austria

**Keywords:** Bioethanol, Xylose fermentation, *Candida tenuis* CBS4435, BP000, Kinetic modeling, Metabolic control analysis, Metabolite profiling

## Abstract

**Background:**

Unlike xylose-converting natural yeasts, recombinant strains of *Saccharomyces cerevisiae* expressing the same xylose assimilation pathway produce under anaerobic conditions xylitol rather than ethanol from xylose at low specific xylose conversion rates. Despite intense research efforts over the last two decades, differences in these phenotypes cannot be explained by current metabolic and kinetic models. To improve our understanding how metabolic flux of xylose carbon to ethanol is controlled, we developed a novel kinetic model based on enzyme mechanisms and applied quantitative metabolite profiling together with enzyme activity analysis to study xylose-to-ethanol metabolisms of *Candida tenuis* CBS4435 (*q*_xylose_ = 0.10 g/g_dc_/h, 25 °C; *Y*_ethanol_ = 0.44 g/g; *Y*_xylitol_ = 0.09 g/g) and the recombinant *S. cerevisiae* strain BP000 (*q*_xylose_ = 0.07 g/g_dc_/h, 30 °C; *Y*_ethanol_ = 0.24 g/g; *Y*_xylitol_ = 0.43 g/g), both expressing the same xylose reductase (XR), comprehensively.

**Results:**

Results from strain-to-strain metabolic control analysis indicated that activity levels of XR and the maximal flux capacity of the upper glycolysis (UG; both ≥ tenfold higher in CBS4435) contributed predominantly to phenotype differentiation while reactions from the oxidative pentose phosphate pathway played minor roles. Intracellular metabolite profiles supported results obtained from kinetic modeling and indicated a positive correlation between pool sizes of UG metabolites and carbon flux through the UG. For CBS4435, fast carbon flux through the UG could be associated with an allosteric control of 6-phosphofructokinase (PFK) activity by fructose 6-phosphate. The ability of CBS4435 to keep UG metabolites at high levels could be explained by low glycerol 3-phosphate phosphatase (GPP, 17-fold lower in CBS4435) and high XR activities.

**Conclusions:**

By applying a systems biology approach in which we combined results obtained from metabolic control analysis based on kinetic modeling with data obtained from quantitative metabolite profiling and enzyme activity analyses, we could provide new insights into metabolic and kinetic interactions contributing to the control of carbon flux from xylose to ethanol. Supported by evidences presented two new targets, PFK and GPP, could be identified that aside from XR play pivotal roles in phenotype differentiation. Design of efficient and fast microbial ethanol producers in the future can certainly benefit from results presented in this study.

**Electronic supplementary material:**

The online version of this article (doi:10.1186/s13068-015-0340-x) contains supplementary material, which is available to authorized users.

## Background

**Fig. 1:**
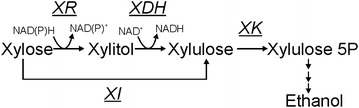
Xylose assimilation pathways addressed by metabolic engineering of *S. cerevisiae*. XR, XDH, XK and XI denote xylose reductase, xylitol dehydrogenase, xylulokinase and xylose isomerase, respectively

Xylose assimilation (XA) represents one of the most intensively studied pathway-engineering problems over the last two decades, with the aim to develop recombinant strains of *Saccharomyces cerevisiae* that are capable of converting xylose, the second most abundant sugar aside from glucose in lignocellulosic materials, efficiently to ethanol. Engineering efforts pursued in the past focused on two routes, namely the bacteria-type and the yeast-type XA pathway [1–7]. Unlike bacteria and some fungi which directly convert xylose to xylulose catalyzed by xylose isomerase, xylose-utilizing yeasts perform this isomerization in two consecutive steps, via xylitol, catalyzed by an NAD(P)H-dependent xylose reductase (XR) and an NAD^+^-specific xylitol dehydrogenase (XDH) (Fig. 1). Resultant xylulose is converted into xylulose 5-P (X5P) by an ATP-dependent xylulokinase (XK) and X5P is further metabolized via reactions of the pentose phosphate (PP) pathway and glycolysis to ethanol.

In the past construction of stable *S. cerevisiae*, constructs that were able to ferment xylose to ethanol were typically based on chromosomal integration of genes encoding for wild-type forms of XR and XDH (frequently from *Scheffersomyces stipitis*) together with an XK gene and with each gene under the control of a constitutive promoter. However, compared to their natural source strains which express the same XA pathway, these strain constructs display lower xylose conversion rates (*q*_xylose_) and produce xylitol (*Y*_xylitol_ = 0.29–0.59 g/g (gram xylitol formed per gram xylose utilized); 0.0–0.1 g/g for *S. stipitis*) rather than ethanol (*Y*_ethanol_ ~ 0.25 g/g; ~0.40 g/g for *S. stipitis*) [8–10]. Opposite metabolic phenotypes should be a direct consequence of different flux control because recombinant and natural yeasts use the same reaction network to convert xylose to ethanol. Various hypotheses about possible targets which exert control on *Y*_xylitol_ and *q*_xylose_ are discussed in the literature. Formation of large amounts of xylitol by recombinant *S. cerevisiae* strains was associated with unbalanced coenzyme usage between XR and XDH [7, 11]. Low *q*_xylose_ was attributed to limiting enzyme activity levels of the XA pathway [12, 13], the PP pathway [14] or xylose uptake [15]. Steady-state kinetic modeling supported by experimental data was used to suggest that a low activity ratio for XR-to-XDH combined with a moderately expressed XK could be essential in keeping *Y*_xylitol_ low [12, 16, 17]. Selective overexpression of XR from *S. stipitis* (SsXR) resulted in low *Y*_xylitol_ and fast xylose conversion but had no effect on *Y*_ethanol_, while glycerol [due to dihydroxyacetone-P (DHAP) reduction activity of SsXR] and acetate were formed instead [13]. Furthermore, it was recognized that increasing cellular availability of NADH for XR by altering redox metabolism decreases *Y*_xylitol_ and *q*_xylose_ while increasing cytosolic NADPH availability leads to faster xylose conversion but also enhanced *Y*_xylitol_ [18, 19]. In contrast to these observations, natural xylose-converting yeasts manage to balance coenzyme usage between XR and XDH and ferment xylose efficiently, despite a XR-to-XDH activity ratio of >1 [9].

With the aim to improve our understanding of how kinetic interactions between XA pathway enzymes and reactions of the central carbon metabolism control *Y*_xylitol_ and *q*_xylose_, we developed an enzyme-mechanism-based kinetic model and analyzed kinetic control schemes of xylose fermentations of the xylose-converting yeast *Candida tenuis* CBS4435 and of its recombinant counterpart, *S. cerevisiae* BP000; we recorded profiles of intracellular metabolites and specific enzyme activities to corroborate results obtained from in silico analyses. Finally, we used the obtained kinetic model of BP000 to predict effects on *Y*_xylitol_ and *q*_xylose_ due to selective modifications in the metabolic network.

Both strains used in our study express a yeast-type XA pathway that involves the same XR (CtXR), an NAD^+^-specific XDH and an XK. Construction, physiological and metabolomic characterization of BP000 have been reported recently [20, 21]. Under anaerobic conditions, BP000 converts 40 % of xylose carbon to xylitol and 24 % to ethanol. The physiology of CBS4435 when growing on xylose has previously been analyzed under aerobic conditions [22] and was assessed for the first time under anaerobic conditions in our study.

## Results

### Xylose fermentations

**Fig. 2:**
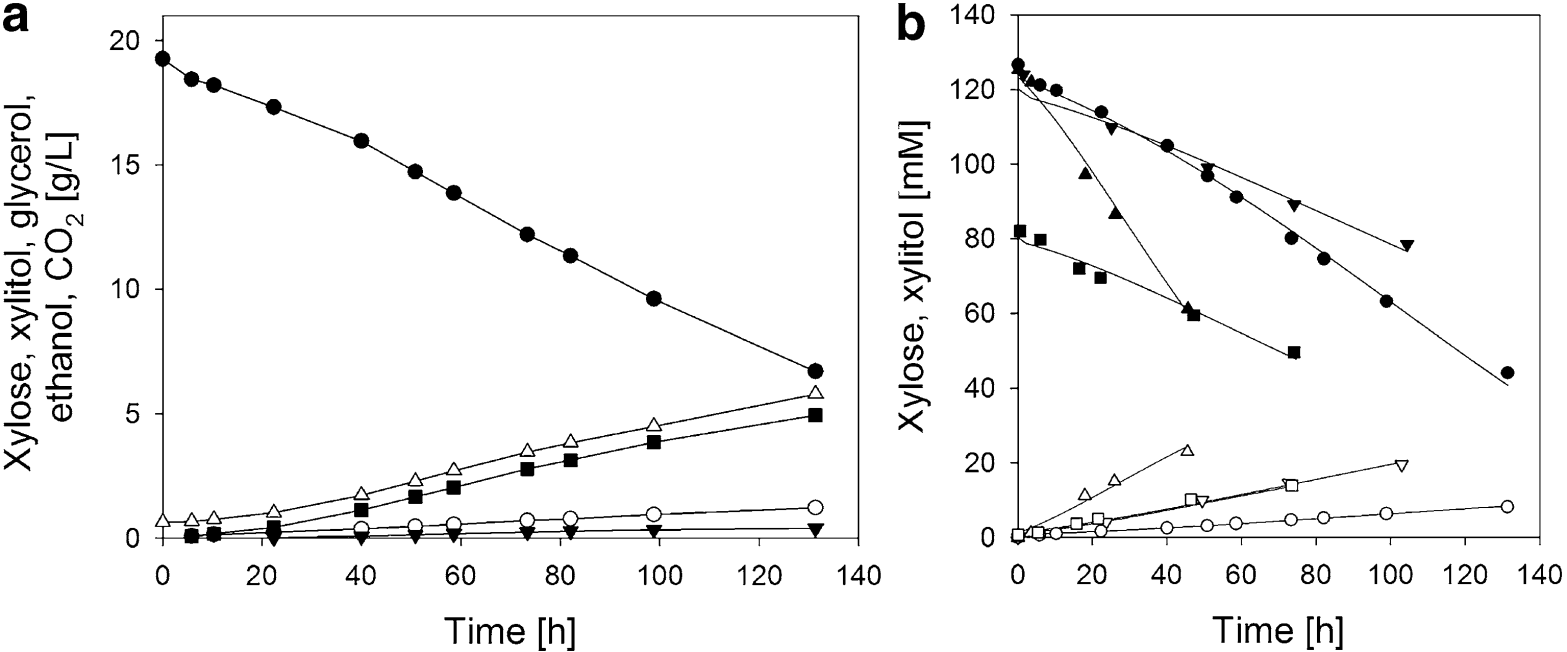
Representative substrate and product time courses of xylose-to-ethanol fermentation obtained for CBS4435 in a bioreactor under anaerobic conditions (**a**) and representative results from parameter estimation analysis (**b**). **a** *Full circles* xylose, *empty circles* xylitol, *empty triangles* ethanol, *full squares* CO_2_ (g/L CO_2_ produced), *full triangles* glycerol. **b** *Full* (*empty*) *symbols* indicate xylose (xylitol) concentrations. Fermentations were carried out with CBS4435 (*circles*) and BP000 [this study: biomass loading: 0.9 g_dc_/L, 18 g/L xylose (*triangles down*), 3.8 g_dc_/L, 18 g/L xylose (*triangles up*) and data from Ref. [20]: 12 g/L xylose and 1.6 g_dc_/L (*squares*)]. *Solid lines* indicate best fits obtained from parameter estimation analysis

Fermentations for CBS4435 and BP000 were carried out in a stirred bioreactor under anaerobic conditions at controlled pH and temperature. For CBS4435, representative time courses of substrate utilization and product formation are shown in Fig. 2a. Physiological parameters obtained for CBS4435 and BP000 are summarized in Table 1. Results obtained for BP000 in this study were in excellent agreement with those reported recently [21]. Similar to BP000, CBS4435 did not grow under these conditions. In contrast to BP000, CBS4435 was able to convert xylose to ethanol at very high yields of 0.44 g/g. Only small amounts of xylitol (0.09 g/g) and glycerol (0.04 g/g) were formed in addition. The *q*_xylose_ for CBS4435 recorded at 25 °C was 1.5-fold higher than that measured for BP000 at 30 °C. Consequently, about 2.5 times more ethanol was formed per g_dc_ and hour by CBS4435.

**Table 1:** Summary of product yields and specific xylose conversion rates obtained for BP000 and *C. tenuis* CBS4435

	BP000	CBS4435
Cell loading (g_dc_/L)	0.9	1.1
*q*_xylose_ (g/g_dc_/h)	0.07 ± 0.01	0.10 ± 0.01

	Yield (g/g)	Yield (g/g)
Xylitol	0.43 ± 0.01	0.09 ± 0.01
Glycerol	0.05 ± 0.01	0.04 ± 0.02
Ethanol	0.24 ± 0.01	0.44 ± 0.02
CO_2_	0.27 ± 0.03	0.42 ± 0.04
Acetate	0.04 ± 0.01	n.d.
Mass balance	1.03 ± 0.04	0.99 ± 0.05

*n.d.* Not detectable

**Fig. 3:**
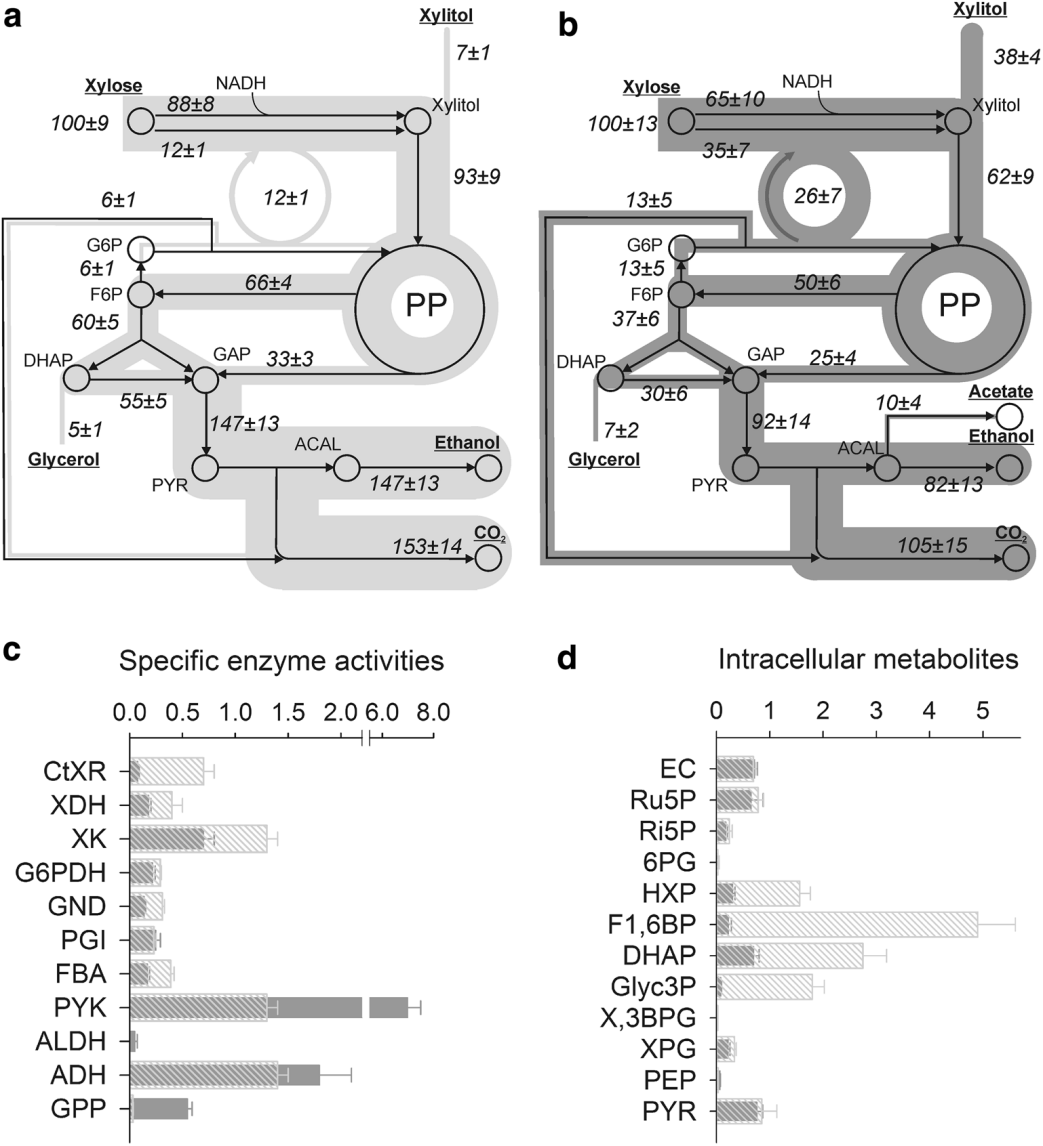
Metabolic flux maps of xylose-to-ethanol fermentation for CBS4435 (**a**) and BP000 (**b**) together with a collection of specific enzyme activities (**c**) and intracellular metabolites (**d**) obtained for CBS4435 (*hatched boxes*) and BP000 (*grey boxes*). **a**, **b** External metabolites are *underlined*. Relative fluxes normalized to *q*_xylose_ (0.67 mmol/g_dc_/h for CBS4435 and of 0.47 mmol/g_dc_/h for BP000) are shown. ATP and CO_2_ formation were used individually as objective function; resultant fluxes were averaged and presented together with their standard deviations. **c** Specific enzyme activities determined at 25 °C are shown in µmol/min/mg_protein_. **d** Intracellular metabolites are shown in µmol/g_dc_. *EC* energy charge

Physiological parameters were further verified by flux balance analysis carried out under experimentally obtained boundary conditions. Resultant flux maps are shown in Fig. 3a, b, respectively. In CBS4435, CtXR reduced 88 % of xylose with NADH while only 65 % was converted by NADH in BP000. In BP000, the ~threefold higher NADPH usage of CtXR compared to CBS4435 was met to 75 % by a 2.2-fold larger flux through the oxidative pentose phosphate (oPP) pathway relative to *q*_xylose_ and to 25 % by the production of acetate. In BP000, 26 % of xylose carbon entering glycolysis at the F6P node were shuttled through the oPP pathway while in CBS4435 only 9 % entered the same pathway.

### Analysis of enzyme activities

Specific activities of XR, XDH, XK, G6PDH, GND, PGI, GPP, ALDH, ADH, FBA, PYK and PFK were determined from cell-free extracts of BP000 and CBS4435 (Fig. 3c, Additional file 1: Table S1). Largest differences were observed for specific activity levels of GPP (17-fold higher in BP000), CtXR (ninefold higher in CBS4435) and PYK (sevenfold higher in BP000), while the remaining enzyme activities were either approx. twofold higher in CBS4435 (XDH, XK, GND and FBA), very similar for both strains (PGI, G6PDH, ADH) or only measureable in one strain (NADP^+^-dependent ALDH and PFK). No activities were observed for NAD^+^-dependent ALDH in either strain. Lack of ALDH activity supported the observed phenotype of CBS4435 which did not form acetate under the conditions investigated. In case of PFK, we were not able to prepare cell-free extracts for CBS4435 containing active forms.

As a consequence of the ninefold higher specific activity level of CtXR, the XR-to-XDH activity ratio was normal for CBS4435 (1.8) but low for BP000 (0.4). The XR-to-XK activity ratios were low for CBS4435 (~0.5) and BP000 (~0.1).

### Intracellular metabolite profiling

Intracellular metabolites from the glycolysis, PP pathway as well as energy metabolites were quantitatively analyzed for BP000 and CBS4435 metabolizing xylose at the pseudo-steady state of conversion (Fig. 3d). For both strains, the physiological state, “metabolizing”, was reflected by an energy charge of ~0.7 [21]. F1, 6BP (21 ± 6-fold), Glyc3P (20 ± 3-fold), HXP (5 ± 1-fold) and DHAP (4 ± 1-fold) were the only intracellular metabolites that showed substantially different levels and were higher in CBS4435 relative to BP000.

### In silico analyses

**Fig. 4:**
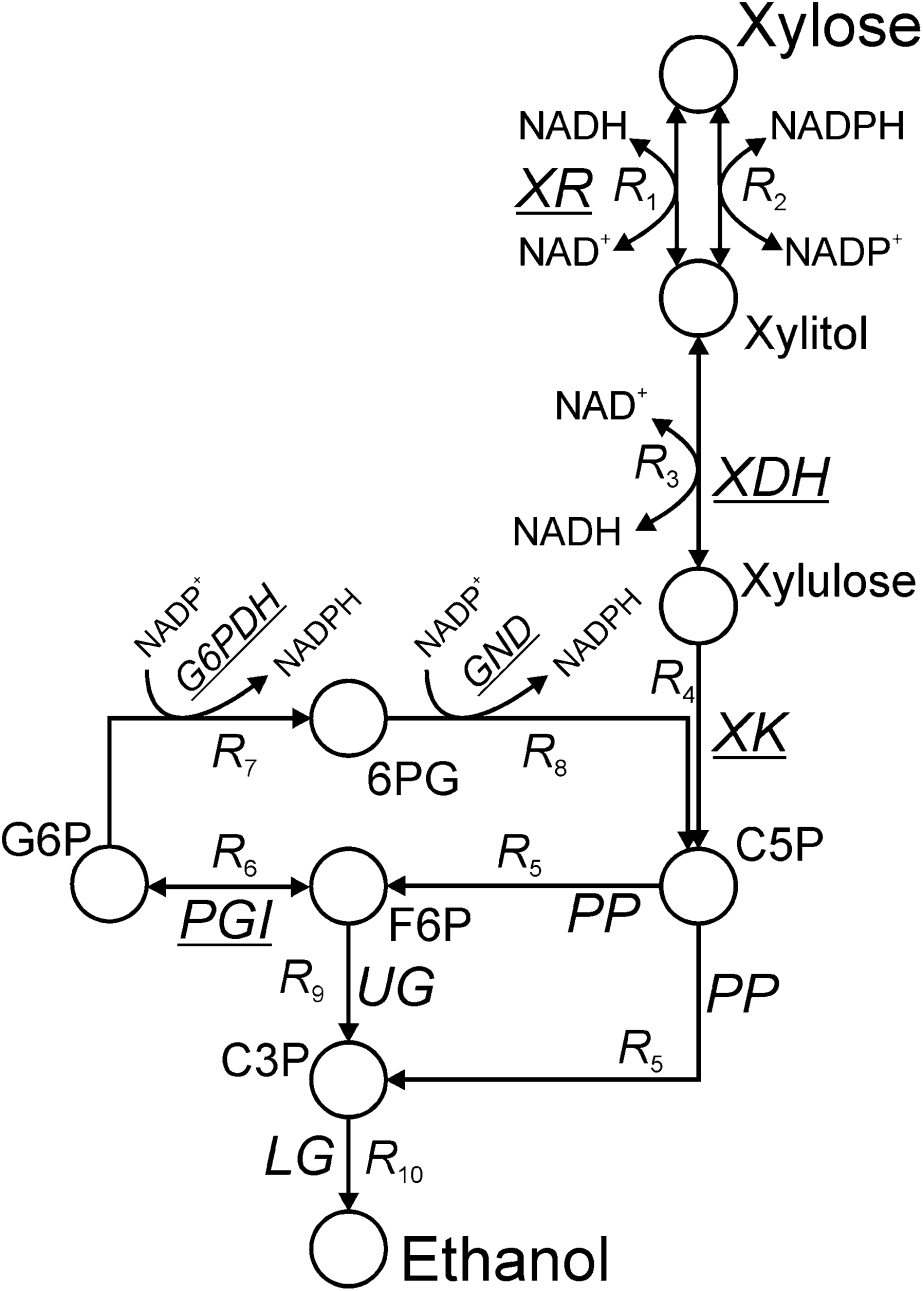
Reaction scheme of the kinetic model used in this work. Reversible reactions (*R*_1_, *R*_2_, *R*_3_, *R*_6_) are indicated by *double arrows* and enzymes are *underlined*. C3P and C5P refer to triose phosphates and pentose phosphates, respectively

To establish the basis for metabolic control analysis (MCA), time courses of xylose utilization and xylitol production recorded for BP000 (18 g/L xylose and 0.9g_dc_/L) and CBS4435 (18 g/L and 1.1 g_dc_/L) were simultaneously fitted four times by parameter estimation analysis by applying the reaction network shown in Fig. 4 as well as rate equations and kinetic parameters collected in the Additional file 1: Table S2 and Table 2, respectively. Resultant best fits (Fig. 2b) showed excellent correlation to experimental data obtained for BP000 (*R*^2^ for *q*_xylose_ and *Y*_xylitol_ were >0.9993 and >0.998, respectively) and CBS4435 (*R*^2^ for *q*_xylose_ and *Y*_xylitol_ were >0.9996 and >0.9991, respectively). Averages and standard deviations obtained for estimated *V*_max_ values are listed in the Additional file 1: Table S3. Enzyme activity levels of restricted reactions and UG varied between ±(0–12) % (BP000) and ±(0–10) % (CBS4435), while enzyme activity levels of PP (BP000: ±86 %; CBS4435: ±69 %) and LG (BP000: ±118 %; CBS4435: ±53 %) showed strong variation. When the kinetic model of BP000 reached steady-state with respect to *q*_xylose_ and *Y*_xylitol_, intracellular concentrations of NAD^+^, NADH, NADP^+^ and NADPH obtained for BP000 by the model (0.62 ± 0.01, 0.30 ± 0.01, 0.020 ± 0.003, 0.011 ± 0.003 mM, respectively) were close to those experimentally obtained (0.5, 0.3, 0.03, 0.02 mM, respectively) for the same strain under comparable conditions in a recent study [21].

**Table 2:** Reaction-specific kinetic parameters used in kinetic modeling

CtXR^a^	NAD(H) (*R*_1_)	NADP(H) (*R*_2_)
E	PO^b^	PO^b^
*V*_f_	1.0	1.3
*V*_f_/*V*_r_	13	25
*K*_iA_ (mM)	0.18 ± 0.04	0.0019 ± 0.0001^c^
*K*_mA_ (mM)	0.04 ± 0.01	0.005 ± 0.002
*K*_mB_ (mM)	98 ± 22	108 ± 17
*K*_iB_ (mM)^d^	392	3920
*K*_iQ_ (mM)	0.344 ± 0.03	0.0059 ± 0.0004^c^
*K*_mQ_ (mM)	0.019 ± 0.005	0.008 ± 0.001
*K*_mP_ (mM)	529 ± 34	220 ± 18
*K*_iP_ (mM)	758 ± 48	556 ± 48
*K*_eq_^Haldane, e^	134	142
*K*_eq_^exp, e^	142	156

GmXDH^f^(*R*_3_)		PP (*R*_5_), UG (*R*_9_), LG (*R*_10_)	
E	PO^b^	*V*_f_	PO^b^
*V*_f_	1.0	*K*_m_ (mM)	0.1
*V*_r_	12.6		
${K_{\rm{m,NAD}^{+}}}$ (mM)	0.14	PGI^h^ (*R*_6_)	
*K*_m,xylitol_ (mM)	14	*V*_f_	PO^b^
${K_{\rm{i,NAD}^{+}}}$ (mM)	0.78	*K*_m,F6P_ (mM)	0.15
*K*_i,xylitol_ (mM)^d^	4.5	*K*_m,G6P_ (mM)	0.30
*K*_i,NADH_ (mM)	0.025	*K*_eq_	3.23
*K*_m,NADH_ (mM)	0.073		
*K*_m,xylulose_ (mM)	4.0	G6PDH (*R*_7_)	
*K*_i,xylulose_ (mM)^g^	1.0	*V*_f_	PO^b^
*K*_eq_^Haldane, e^	7 × 10^−4^	${K_{\rm{m,NADP}^{+}}}$ (mM)	0.1
*K*_eq_^exp, e^	(4-7) × 10^−4^	*K*_m,G6P_^h^ (mM)	0.05
		*K*_i,NADPH_ (mM)	0.20 (0.05)^i^
XK (*R*_4_)			
*V*_f_	PO^b^	GND (*R*_8_)	
*K*_m,xylulose_ (mM)^h^	0.31	*V*_f_	PO^b^
		*K*_m,NADP_^+^ (mM)	0.02
		*K*_m,6PG_^h^ (mM)	0.05
		*K*_i,NADPH_ (mM)	0.02

^a^A, B, P, Q relate to NAD(P)H, xylose, xylitol, and NAD(P)^+^, respectively

^b^PO, values were found within a predefined range by parameter optimization (see main text and Additional file 1: Table S3)

^c^Values obtained from ligand-binding analysis using fluorescence spectroscopy (this study)

^d^Values calculated from Haldane relationship *K*_eq_ = *V*_f_^2^ *K*_iP_ *K*_mQ_/(*V*_r_^2^ *K*_iB_ *K*_mA_) [38]

^e^Equilibrium constants were calculated in accordance with the Haldane relationship [*K*_eq_^Haldane^ = *V*_f_ *K*_mP_ *K*_iQ_/(*V*_r_ *K*_mB_ *K*_iA_)] [38]. Values can be compared to those experimentally obtained in this study (CtXR) or to reported *K*_eq_^exp^ [39, 40]. Calculated Gibbs free energies of reaction of 6 ± 1 kJ mol^−1^ for the isomerization of xylose into xylulose [Δ*G*_r_ = −RTln(*K*_eqXR_^Haldane^ *K*_eqXDH_^Haldane^] or Δ*G*_r_ = −RTln(*K*_eqXR_^exp^ *K*_eqXDH_^exp^) are in excellence accordance with a value of 4.3 kJ mol^−1^ calculated from standard transformed Gibbs free energies of formation [41]

^f^Reported kinetic parameters obtained from comprehensive full-kinetic study acquired at 25 °C in 50 mM potassium phosphate buffer pH 7.5 were applied [42]. A, B, P, and Q correspond to NAD^+^, xylitol, xylulose, and NADH, respectively. Note, to fulfill thermodynamic with respect to *K*_eq_^exp^ reported upper limits were used for *V*_r_ (1800 ± 350 s^−1^), *K*_B_ (12 ± 2 mM) and lower limits for *K*_P_ (8 ± 4 mM)

^g^Value was taken from [43]

^h^Values of kinetic parameters for XK, G6PDH, 6PGDH, and PGI were from Refs. [33, 44–46], respectively

^i^Value referred to CBS4435 (BP000). Note based on a sensitivity analysis implemented in Copasi *K*_i_ of both ScG6PDH and CtG6PDH did not significantly influence FCC and YCC

**Fig. 5:**
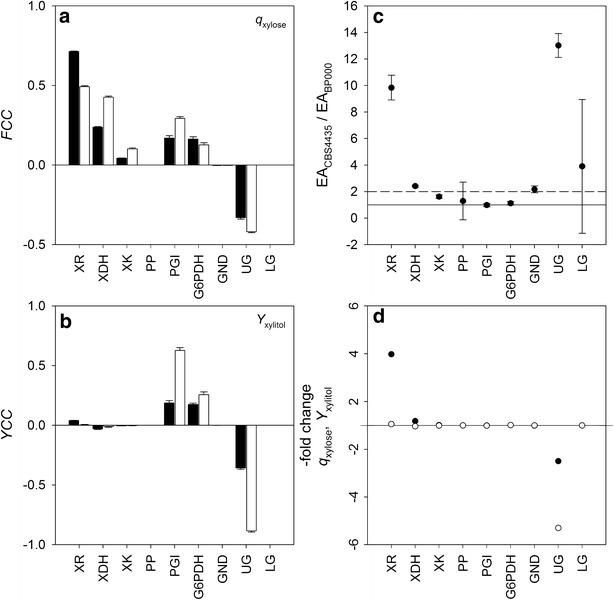
Results from traditional MCA (**a**, **b**) and strain-to-strain MCA (**c**, **d**). **a**, **b** Display flux control coefficients (FCC) for *q*_xylose_ and yield control coefficients (YCC) for *Y*_xylitol_, respectively. Note, in accordance with MCA theory FCC and YCC sum up to one (0.99 ± 0.02) and zero (0.01 ± 0.03), respectively [23]. *Black* and *white bars* represent data obtained for BP000 and CBS4435, respectively. **c** Shows the ratio of activity levels obtained for each reaction of CBS4435 (EA_CBS4435_) and BP000 (EA_BP000_) by parameter estimation analysis. *Solid* and *dashed lines* indicate ratios of 1 and 2, respectively. **d** Shows -fold changes on *q*_xylose_ (*full circles*) and *Y*_xylitol_ (*empty circles*) of BP000 due to individual changes of activity levels from the level obtained for BP000 (EA_BP000_) to the level obtained for CBS4435 (EA_CBS4435_). A ratio of 1 is indicated by the *solid line*. Positive (negative) values in **a**–**d** indicate an *x-*fold higher (lower) value relative to the reference value. Kinetic model obtained for BP000 was used as a reference in **a**–**d**

To determine how activity levels of XR, XDH, XK, PP, PGI, G6PDH, GND, UG and LG influence *Y*_xylitol_ and *q*_xylose_, respective yield (YCC) and flux (FCC) control coefficients, respectively, were determined based on MCA theory and assuming a change in each activity level of 1 % [23]. These traditional MCAs were performed for each strain and averages obtained at the steady state for FCC and YCC are shown in Fig. 5a, b, respectively. Note that a positive and negative control coefficient imply that an increase of activity level relative to its reference value is accompanied by an increase and decrease of *q*_xylose_ or *Y*_xylitol_ relative to its reference value, respectively. There is no control on conversion rate or yield when the control coefficient is 0. Both strains exhibited similar control patterns for *q*_xylose_ (positive FCC: XR > XDH > PGI ≥ G6PDH > XDH; negative FCC: UG; no control on *q*_xylose_: PP, GND, LG) and *Y*_xylitol_ (positive YCC: PGI ≥ G6PDH ≫ XR; negative YCC: UG ≫ XDH > XK; no control on *Y*_xylitol_: PP, GND, LG). FCCs and YCCs of G6PDH, GND, PGI and PP obtained for BP000 were in good agreement with previous experimental findings [14, 18, 19].

A comparison of activity level estimates obtained by parameter estimation analysis for BP000 and CBS4435 quantitatively corroborated trends that were observed in the enzyme activity analysis. Activity level estimates for UG were 13 ± 1-fold higher for CBS4435, while those of PP and UG were not distinguishable (Fig. 5c). To identify those reactions contributing to phenotype differences between BP000 and CBS4435, a strain-to-strain MCA was carried out. To this end, each activity level estimate obtained for BP000 was set individually to that value obtained for CBS4435 and effects on *q*_xylose_ and *Y*_xylitol_ were determined. Results are shown in Fig. 5d. Of the nine reactions tested, only three affected *Y*_xylitol_ and/or *q*_xylose_ significantly. Individual changes of XR (ninefold) and XDH (2.4-fold) activity levels led to a fourfold and 1.2-fold increase of *q*_xylose_, respectively, while changing the activity level of UG (13-fold) resulted in large decreases of both *q*_xylose_ (2.5-fold) and *Y*_xylitol_ (5.3-fold). UG represents a novel target involved in the control of xylose-to-ethanol conversion in yeasts. Note that an increase of both XR and UG activity levels is required to switch from a BP000-like phenotype to a CBS4435-like phenotype.

**Fig. 6:**
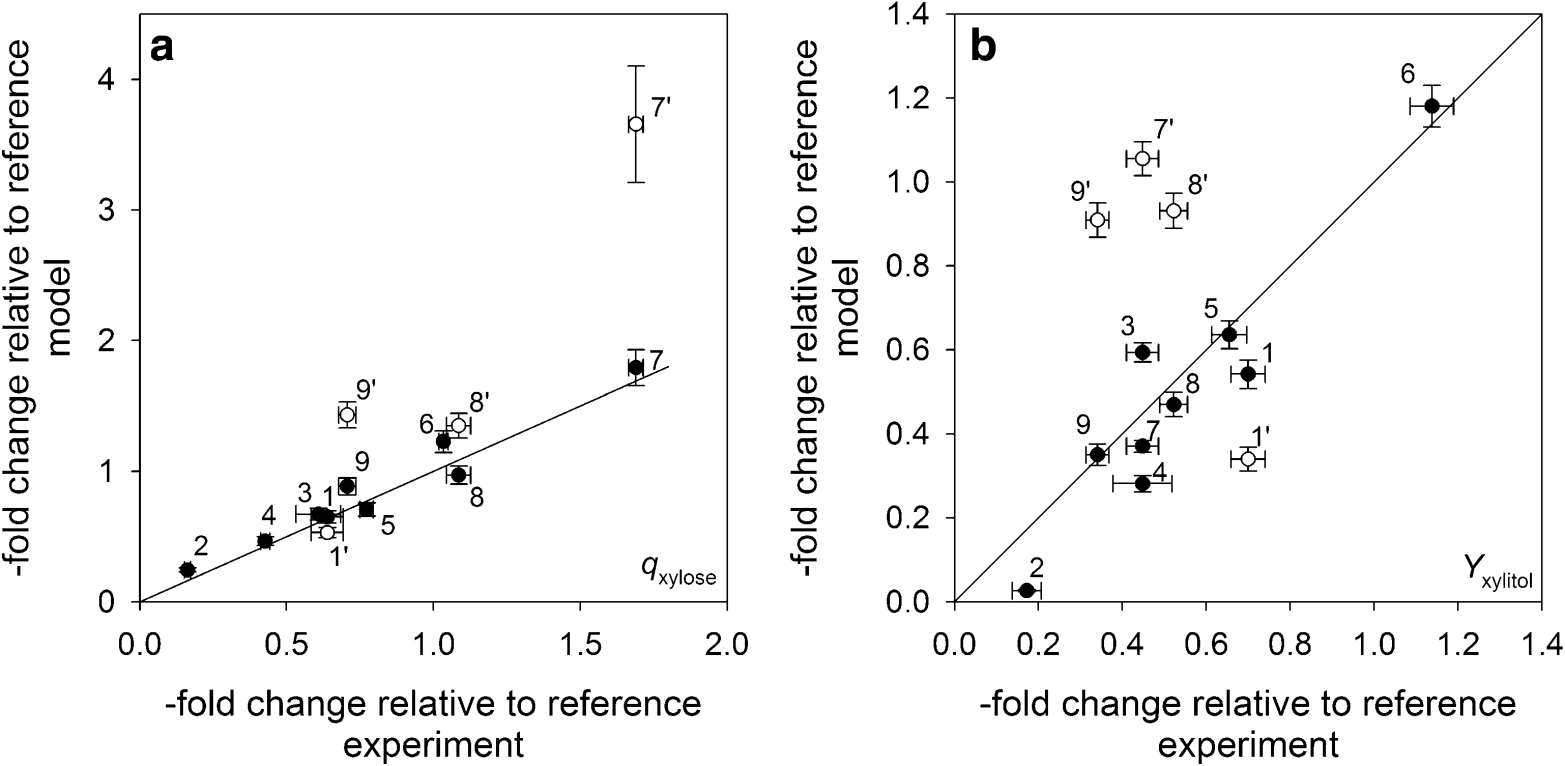
Results from predictability analysis. **a**, **b** Display comparisons of predicted to experimentally observed effects on *q*_xylose_ and *Y*_xylitol_, respectively, relative to a reference state due to different network modification (see main text). Numbering used: *1* and *1′* fivefold and tenfold lower activity levels of PGI; *2*, *4*, *5*, and *6* knockout, 20-fold and fivefold lower and 5.6-fold higher activity levels of G6PDH; *3* knockout of GND; *7′* (6.7–10)-fold higher activity levels of XR; *8′* and *9′* 29-fold and 72-fold higher activity levels of XDH. Data *7*, *8* and *9* display corresponding effects after increasing the activity level of UG by a factor of 6 (XR: tenfold higher), *4* and *6*, respectively. *Solid lines* indicate perfect match of simulated vs. experimentally obtained effects. To simulate knockouts, a specific activity of 0.001 µmol/min/mg^−1^ was assumed (a value <0.01 µmol/min/mg has been reported [18]). *Error bars* indicate reported and obtained, by in silico analysis (±10 % variation on the extent of activity level modification was assumed), standard deviations

To test whether the kinetic model can be used to predict changes in *Y*_xylitol_ and *q*_xylose_ due to a change in the activity levels of one network reaction, we applied data from the literature. Results for *q*_xylose_ and *Y*_xylitol_ are shown in Fig. 6a, b respectively. Enzyme activity level estimates obtained for BP000, which displays a genotype and phenotype that is comparable to the reference strains used in these studies, served as a basis to simulate reduction of PGI activity (modifications 1 and 1′ [18]), knockout of GND (modification 3 [18]), knockout and variation of G6PDH activity levels (modifications 2, 4–6 [18, 19]), and increase of XR (modifications 7 and 7′ [13]) or XDH (modifications 8, 8′, 9, and 9′ [17]) activity levels. The experimentally observed effects for altering G6PDH and GND levels on *Y*_xylitol_ and *q*_xylose_ could be quantitatively predicted. Only a small adaption of the PGI activity level (reduction by a factor of five instead of ten as reported [18]) was needed to match reported effects on conversion rate and xylitol yield. However, when XR or XDH activity levels were individually addressed effects on *q*_xylose_ and *Y*_xylitol_ could not be predicted. *V*_max_ of UG had to be increased in addition by a factor of 6 (XR), 4 (XDH, modification 8) and 6 (XDH, modification 9) to simulate reported effects. Results, therefore, pointed to a more complex substrate-to-enzyme reaction rate relationship for UG.

Additional parameter estimation analyses were performed to challenge the kinetic model with respect to biomass load and initial xylose concentrations. Experimental data obtained in our study (3.8 g_dc_/L BP000, 18 g/L xylose) and from a recent study by our workgroup [20] (1.6 g_dc_/L BP000, 12 g/L xylose) were fitted as described above and results are summarized in the Additional file 1: Table S3. Resultant models fitted time courses of xylose utilization and xylitol formation with *R*^2^ > 0.999 (*q*_xylose_) and *R*^2^ > 0.988 (*Y*_xylitol_) (Fig. 2b). Activity levels obtained from in silico analysis for all three BP000 xylose fermentations were identical with regard to the average verifying the usability and robustness of the kinetic model (Additional file 2: Fig. S1).

## Discussion

### *Candida tenuis* CBS4435 a potential alternative to current xylose-fermenting yeasts

Even in the first microorganism screenings, the yeast strain *C. tenuis* CBS4435 was recognized as a potential candidate for production of ethanol from xylose [24, 25]. Interestingly, however, no further studies have yet been performed with this strain in this respect. In our study, we quantitatively evaluated the capability of this CBS4435 strain to ferment xylose with respect to physiological parameters and compared it to its recombinant derivative BP000. Based on the key process parameters *Y*_ethanol_ (0.44 g/g), *Y*_xylitol_ (0.1 g/g) and *q*_xylose_ (0.1 g/g_dc_/h) obtained in this study, *C. tenuis* CBS4435 can be ranked together with known *S. stipitis* strains among the best xylose-utilizing native yeasts [8].

### Factors controlling strain fitness of natural and recombinant xylose-converting yeasts

There is an ongoing discussion about the difference in metabolic phenotypes between xylose-converting natural yeasts which efficiently ferment xylose to ethanol relative to their recombinant derivatives of *S. cerevisiae* which express the same XA pathway but produce xylitol instead of ethanol at low conversion rates. To address this question, we used *C. tenuis* CBS4435 and *S. cerevisiae* BP000 as representatives of natural and recombinant xylose-converting yeasts, respectively, and used a systems biology approach. We started with a kinetic model that is based on in vitro determined enzyme kinetic data to identify, by applying metabolic control analysis, candidate reactions that likely enable CBS4435 to ferment xylose efficiently. In the next step, results obtained from in silico analysis were verified by quantitative metabolite profiling and enzyme activity analysis. Finally, a metabolic scenario was developed with which differences in the metabolic phenotypes could be explained.

To identify target reactions that control *q*_xylose_ and *Y*_xylitol_, we developed a new kinetic model and performed parameter estimation analysis using time courses of xylose utilization and xylitol formation as basis. In contrast to other enzyme mechanism-based kinetic models that have been used to study the yeast-type XA pathway in the past [16, 26], in our study XR utilized both coenzymes, NADPH was regenerated by reactions of the oPP pathway connected to the network, reactant concentrations were not fixed, the XA pathway was connected to the central carbon metabolism and in silico analyses were performed based on experimentally obtained time courses instead of assumed steady states. Parameter estimation analysis showed that enzyme activity levels and enzyme kinetic parameters measured by standard in vitro assays are highly suitable to describe time-dependent utilization of xylose and formation of xylitol for both BP000 and CBS4435. This observation suggests that xylose uptake did not limit *q*_xylose_ in either strain. We were able to demonstrate that the kinetic model was robust to changes in biomass loads, initial xylose concentrations and selective network modifications in the central carbon metabolism. However, effects on *q*_xylose_ and *Y*_xylitol_ caused by increasing XR or XDH activity levels were merely simulated rather than predicted by the kinetic model. Results from MCA and the predictability study led to the suggestion that in contrast to network modifications executed downstream from the XA pathway, overexpression of XR or XDH positively affected the maximal flux capacity of UG. Underlying reasons at the molecular level are not known but our results indicated that the maximal flux capacity of UG is allosterically controlled in xylose-utilizing recombinant *S. cerevisiae*.

Since traditional MCA of kinetic models obtained for BP000 and CBS4435 provided no clue about targets involved in phenotype differentiation, we carried out MCA that was based on a strain-to-strain comparison and found two major targets, the XR activity level (tenfold higher in CBS4435) and the maximal flux capacity of UG (13-fold higher in CBS4435) and one minor target, the XDH activity level (twofold higher in CBS4435). Hence, aside from small positive contributions made by XDH to *q*_xylose_, high activity levels of XR paired with high flux capacities of UG are essential for CBS4435 to keep *Y*_xylitol_ at low levels while permitting fast utilization of xylose relative to that of BP000. The resulting 2.3-fold [=0.67 × 0.60/(0.47 × 0.37)] faster carbon flux at the F6P node towards C3-carbon phosphates is, therefore, a consequence of a kinetic pull exerted by reactions of the UG rather than by blocking carbon flux through reactions of the oPP pathway. The 13-fold higher *V*_max_ of UG predicted by the kinetic model for CBS4435 cannot be explained by the twofold higher levels of FBA observed for CBS4435. Only little flux control by FBA can be assumed as the reaction operates close to equilibrium [21]. Although we were not able to measure PFK activity in CBS4435 cell-free extracts, our results from strain-to-strain MCA, quantitative metabolite profiling and enzyme activity analysis provided sufficient indirect evidence to deduce an essential role of PFK in controlling carbon flux through UG. In a recent study, we showed that in BP000 metabolizing xylose under anaerobic conditions, the PFK activity should be hypersensitive to a change in the F6P level [21]. A five-fold higher F6P level (assuming that PGI reaction is at equilibrium in both strains), as observed here for CBS4435 relative to BP000, should therefore translate into a faster reaction rate of the ScPFK catalyzed reaction. A 9.4-fold increase of PFK activity can be calculated by applying the respective allosteric reaction model and reported reactant concentrations [21, 27]. Although the reaction mechanism of CtPFK is not known, a similar kinetic push by an atypical Michaelis Menten dependence for F6P is conceivable for CtPFK in light of our results obtained in this study.

High XR activity levels (XR-to-XDH >1) in recombinant *S. cerevisiae* strains have always led to an increase of *q*_xylose_, a decrease of *Y*_xylitol_ but also to an enhanced release of glycerol which was explained by the ability of SsXR to reduce DHAP with NADH [13]. On the contrary, the expression at similarly high levels of SsXR in *S. stipitis* [8] and CtXR in CBS4435 [CtXR is also active on DHAP (Additional file 3)] did not induce pronounced glycerol formation even though the Glyc3P level in CBS4435 [high levels of Glyc3P were also found by our workgroup for *S. stipitis* (Additional file 2: Fig. S2)] was about 20-fold higher relative to BP000. Supported by enzyme activity measurements, the accumulation of high Glyc3P levels in CBS4435 was mostly an effect of limiting GPP activity levels. Deletion of both isoenzyme forms of GPP in *S. cerevisiae* resulted in a strain that also accumulated Glyc3P [28]. Based on the presented data, a more general metabolic scenario for efficient conversion of xylose to ethanol is proposed for natural yeasts: carbon flux from F6P towards ethanol is kept at a high rate by maintaining large pool sizes of metabolites of the UG which build up as a consequence of (1) pushing xylose carbon through the PP pathway and (2) blocking the metabolic flux through the glycerol pathway.

## Conclusions

In this work, we could demonstrate that integrating data obtained from metabolic control analysis based on kinetic modeling, quantitative metabolite profiling and enzyme activity analysis represent a powerful approach to identify metabolic and kinetic interactions that play an important role in controlling the key process parameters, *q*_xylose_ and *Y*_xylitol_. With PFK and GPP, two new targets were identified based on the evidences presented. From a metabolic engineering point of view, the proposed control mechanism conducted by these enzymes is interesting as it should be independent of the type of XA. These new insights into the metabolic and kinetic control of *q*_xylose_ and *Y*_xylitol_ will have, therefore, important implications in the future design of efficient and fast microbial ethanol producers.

## Methods

### Microorganisms

*C. tenuis* CBS4435 was obtained from the Centraalbureau voor Schimmelcultures (Baarn, The Netherlands) and maintained at 25 °C on YPD-agar containing yeast extract (1 % w/v), peptone from Casein (2 % w/v), glucose (2 % w/v) and agar (1.6 % w/v). The pH value of the YP portion of the medium was adjusted to 5.5 prior to sterilization. Cells from late-exponential phase grown in YPD medium at 25 °C were used for long-term storage in a glycerol (60 % v/v)—YPD medium at −70 °C. The recombinant *S. cerevisiae* strain BP000 was used that expresses chromosomally integrated XR from *C. tenuis* CBS4435 (CtXR), XDH from *Galactocandida mastotermitis* (GmXDH) and endogenous XK (ScXK), each under the control of a strong constitutive TDH promoter [20].

### Biomass preparation

Biomass of CBS4435 used in conversion experiments was prepared at 25 °C by two consecutive cultivations. First, a loopful of cells was transferred aseptically from a freshly overgrown YPD-agar plate into a baffled 300 mL shake flask containing 50 mL of mineral (M-) medium (see later). Cells were cultivated overnight at 125 rpm. These cultures served as inoculum for the preparation of biomass which was carried out at 125 rpm in baffled 1000 mL shake flasks containing 300 mL of M-medium. The initial optical density at 600 nm (OD_600_) was 0.05. Cells from preparatory cultures were harvested at an OD_600_ of 3–4 by centrifugation (4400*g*, 15 min, 4 °C) and washed once with a cold physiological NaCl (0.9 % w/v) solution prior to xylose conversion experiments. The M-medium used contained 14.4 g/L KH_2_PO_4_, 5 g/L (NH_4_)_2_SO_4_, 0.5 g/L MgSO_4_·7H_2_O, and 0.025 % (v/v) Antifoam 204 (Sigma-Aldrich, Vienna). The pH was adjusted to a value of 5.5 prior to sterilization. Vitamins as well as trace elements were added as described by [29]. Xylose (18 g/L) represented the sole carbon source to induce expression of enzymes which constitute the XA pathway in CBS4435 [22]. The same protocol for biomass preparation was applied for BP000 with the exception of glucose as the carbon source (instead of xylose), an initial pH of 5.5 (instead of 6.5) and a cultivation temperature of 30 °C (instead of 25 °C).

### Anaerobic xylose conversions

Xylose conversions were carried out at 25 °C (CBS4435) or at 30 °C (BP000) in a Labfors bioreactor (Infors HT, Bottmingen, Switzerland, working volume: 2000 mL; stirrer: two six-bladed disc impellers; ratio of impeller to reactor diameter was 0.4; 200 rpm). The same M-medium as described above was used except for KH_2_PO_4_ (3 g/L), ergosterol (10 mg/L) and Tween-80 (0.42 g/L). The pH was maintained at values of 4.5 ± 0.1 (CBS4435) and 5.5 ± 0.1 (BP000). Conversions were carried out with 0.9 and 3.8 g/L (BP000) or with 1.1 g/L (CBS4435) of dry cells. Anaerobic conditions were maintained by sparging the culture continuously with nitrogen (purity 5.0, 0.16 vvm). All conversion experiments were carried out in duplicates.

### Analytics of external metabolites

Sample work-up, monitoring of cell growth (recorded as OD_600_) and off-gas analysis (CO_2_ and ethanol) using an acoustic gas analyzer (IN1313, Innova AirTech Instruments, Ballerup, Denmark) were carried out as described by [29]. Cell dry weights were determined as described recently [30] and correlated with optical densities using an averaged factor of 0.41 g dry cells (g_dc_) per unit of OD_600_ for both strains. Concentrations of xylose and fermentation products were analyzed by HPLC–UV/RI using an Aminex HPX87-H column and 0.5 mM H_2_SO_4_ as eluent [20].

### Analytics of intracellular metabolites

For the analysis of intracellular metabolites, xylose fermentations for BP000 and CBS4435 were carried out in a bioreactor (working volume 1000 mL) as described above and by applying 0.25 g/L KH_2_PO_4_ and 1.3–1.4 g_dc_/L (BP000) or 0.9–1.0g_dc_/L (CBS4435). Low concentrations of KH_2_PO_4_ in the medium were necessary to reduce the effect of ion suppression during LC/MS analysis. The applied KH_2_PO_4_ concentrations did not affect phenotypes of BP000 and CBS4435 (data not shown). Samples were withdrawn after 24 h of xylose fermentation which represented the pseudo-steady state of xylose-to-ethanol conversion. A protocol for sample preparation as described by [21] was used with slight modifications (see Additional file 3). Metabolite samples containing ^13^C-labeled metabolite yeast extract as internal standard (ISTD) were analyzed by LC–MS. A Dionex Ultimate 3000 HPLC setup (Thermo Fisher Scientific, USA) equipped with a reversed-phase Atlantis T3 C18 pre- and analytical column (Waters, USA) was used [31]. For metabolite separation, a 40-min gradient was applied: 2-propanol was used as eluent A and aqueous methanol solution [(5 % methanol *v*/*v*), tributylamine (10 mM), acetic acid (15 mM), pH 4.95] was used as eluent B. The injection volume per sample was 10 µL. Mass spectrometric detection was carried out with an Exactive™ Orbitrap system (Thermo Fisher Scientific, USA). Heated electrospray ionization (HESI) was used for negative ionization and masses between 70 and 1100 *m*/*z* were detected with a resolution of 50,000 (at *m*/*z* 200) in full scan mode. Identical compounds (Sigma-Aldrich, USA, of highest purity available) containing ISTD were used as standards to cover a concentration range of 0.4–100 µM. MS data were analyzed with TraceFinder™-software Version 3.1. (Thermo Fisher Scientific, USA). Eleven intracellular metabolites [ATP, ADP, AMP, ribulose-5P (Ru5P), ribose-5P (Ri5P), 6-phosphogluconate (6PG), fructose 1,6-bisP (F1,6BP), DHAP, glycerol-3P (Glyc3P), phosphoenolpyruvate (PEP) and pyruvate (PYR)] and three metabolite pools [sum of hexose phosphates (HXP), sum of 2PG and 3PG (XPG; PG, phosphoglycerate), sum of 1,3-BPG and 2,3BPG (X,3BPG)] were quantified for each strain and respective concentrations were calculated as averages from a total of eight samples obtained from two individual fermentations (four replicates per fermentation).

### Determination of specific enzyme activities in cell-free extracts

Enzyme activities were analyzed at 25 °C (CBS4435, BP000) and at 30 °C (BP000) from cell-free extracts by means of the initial rate method. Cell-free extracts were prepared at 4 °C from cells metabolizing xylose at the pseudo-steady state using a French Press (1500 psi, SLM Aminco, Silver Spring, MD, USA). Cells were washed once with ice-cold physiological sodium chloride solution and re-suspended in 50 mM potassium phosphate buffer (PBB) pH 7.0 prior to cell disruption. The amount of soluble protein was analyzed using the Bio-Rad Protein Assay (Bio-Rad, Richmond, CA, USA) referenced against bovine serum albumin. Time-dependent utilization of NADPH (XR) or NADH [XR, alcohol dehydrogenase (ADH), XK, phosphoglucose isomerase (PGI), 6-phosphofructokinase (PFK), F1,6BP aldolase (FBA), pyruvate kinase (PYK)] as well as formation of NADH [XDH, aldehyde dehydrogenase (ALDH)] or NADPH (ALDH, G6PDH and GND) was recorded at 340 nm using a DU800 spectrophotometer (Beckman Coulter, Fullerton, CA, USA) equipped with a Peltier temperature controller. A molar extinction coefficient of 6.22/cm/mM was used for NAD(P)H. Release of phosphate from Glyc3P over time was analyzed for Glyc3P phosphatase (GPP) activity. Phosphate concentrations were determined from cleared samples treated at 99 °C for 5 min as described by [32]. Compositions of reaction mixtures are presented in the Additional file 1: Table S4. Appropriate reference measurements were carried out and considered as required.

### Kinetic modeling

The reaction model as shown in Fig. 4 was used as the basis for in silico analysis. To reduce the degree of complexity, stoichiometry of carbon conversion was used for XK (*R*_4_), the PP pathway (*R*_5_), the upper part of glycolysis (UG) (*R*_9_; combining PFK and FBA) and for the lower part of glycolysis (LG) up to ethanol formation (*R*_10_). We did not consider ATP in the model as under the fermentation conditions used the reaction catalyzed by XK should be well saturated by ATP [compare *K*_ATP_ of 0.17 mM and 0.28 mM reported for XKs of *S. cerevisiae* [33] and *S. stipitis* [34], respectively, with intracellular concentrations of ATP estimated for BP000 and other xylose-metabolizing recombinant *S. cerevisiae* strains (3–4 mM) [21]]. As both strains investigated in this study were not able to grow on xylose under the conditions applied, biomass formation was not included in the model. Respective rate equations and corresponding kinetic parameters are shown in the Additional file 1: Table S2 and Table 2, respectively. Known rate equations including reversibility were used for XDH (*R*_3_) and PGI (*R*_6_). The complete rate equation for CtXR involving reversibility as well as alternate binding of NAD(P)(H) (*R*_1_, *R*_2_) was derived in this study and verified (see Additional files 2: Scheme S1; Fig. S3 and 3). Simplified rate equations were applied for XK (*R*_4_), PP pathway (*R*_5_), UG (*R*_9_) and LG (*R*_10_), while representative rate expressions were used for G6PDH (*R*_7_) and GND (*R*_8_) which take competitive product inhibition by NADPH into account.

Selection of known and determination of so far unknown model-relevant kinetic data are described in detail in the Additional file 3. Briefly, all 24 kinetic parameters required for simulating the XR reaction were determined. Values for $K_{\text{m,NADP}}^{+}$ and *K*_i,NADPH_ were measured for Sc(Ct)G6PDH and Sc(Ct)GND. The set of kinetic parameters describing a reversible ordered Bi Bi mechanism reported for GmXDH was applied to both XDHs. Similarly, a reported *K*_m,xylulose_ for ScXK and the set of kinetic parameters reported for ScPGI were used. For lumped reactions *R*_5_, *R*_9_ and *R*_10_, a Michaelis constant of 0.1 mM was assumed.

### Parameter estimation analysis

Time courses of xylose utilization and xylitol production obtained for CBS4435 (this study) and BP000 (this study and from [20]) were used as a basis to fit the kinetic model by parameter estimation. Both time courses which represent mutually independent parameters were fitted simultaneously. The software package Copasi 4.8.35 (http://www.copasi.org [35, 36]) was used. Only the pseudo-steady-state phase of xylose conversion which is free of substrate limitation and ethanol or other unspecific inhibitions was subjected to parameter estimation by evolutionary programming. After transformation, upper and lower bounds of specific enzyme activities measured at 25 °C (CBS4435) and 30 °C (BP000) were applied to the respective cell-specific molar rates (mmol/h/g_dc_) by assuming that on a mass basis the protein content in dry yeast cells accounts for 40 % [37]. Intracellular activity levels of *R*_5_, *R*_9_ and *R*_10_ were used in a range of 0.1–10, 1–100 and 1–100 mmol/h/g_dc_, respectively. Initial concentrations of coenzymes were constrained to ranges reported for BP000 [21]. A complete summary of input parameters is presented in the Additional file 1: Table S3.

### Flux balance analysis

A metabolic reaction network was designed consisting of 32 reactions involving xylose assimilation, PP pathway, oxidative PP pathway (oPP) and glycolysis as well as glycerol, acetate and ethanol production. For XR, one reaction was used for each coenzyme. Upper and lower bounds of experimentally obtained molar production rates of ethanol, xylitol, glycerol and acetate were applied. To capture network flexibility and experimental variations, simulations were carried out at upper and lower bounds and at the average value of *q*_xylose_. Two different objective functions (CO_2_ and ATP formation) were individually applied for linear optimization by linprog (Optimization Toolbox, Matlab R2013b, Mathworks, Inc., USA).

## Additional files

**Additional file 1.** Additional Tables, Tables S1–S4.
**Additional file 2.** Additional Schemes and Figures; Scheme S1, Figures S1–S3.
**Additional file 3.** Additional information; preparation of intracellular metabolites; derivation of the complete rate law describing simultaneous utilization of NAD(P)H by XR; validation of XR rate equation; acquisition of model-relevant kinetic data; activity of CtXR with DHAP.

## Abbreviations

XA: xylose assimilation
XR: xylose reductase
XDH: xylose dehydrogenase
XK: xylulokinase
PP: pentose phosphate
oPP: oxidative PP
FCC: flux control coefficient
YCC: yield control coefficient
MCA: metabolic control analysis
*R*_i_: reaction rate of the *i*th reaction
Sc: *S. cerevisiae*
Ct: *C. tenuis*
Ss: *Scheffersomyces stipitis*
Gm: *Galactocandida mastotermitis*
*Y*: product yield
*q*: specific conversion rate
ADH: alcohol dehydrogenase
ALDH: aldehyde dehydrogenase
UG: upper glycolysis
LG: lower glycolysis
G6P: glucose 6-phosphate
F6P: fructose 6-phosphate
HXP: hexose phosphate
C3P: triose phosphate
C5P: pentose phosphate
GAP: glyceraldehyde 3-phosphate
DHAP: dihydroxyacetone phosphate
PYR: pyruvate
Ru5P: ribulose 5-phosphate
Ri5P: ribose 5-phosphate
F1,6-BP: fructose 1,6-bisphosphate
X,3 BPG: bisphosphoglycerate, X stands for 1- or 2-
XPG: phosphoglycerate, X stands for 2- or 3-
PEP: phosphenolpyruvate
Glyc3P: glycerol 3-phosphate
