# Supplementary material for: Identification of novel metabolic interactions controlling carbon flux from xylose to ethanol in natural and recombinant yeasts
Source: Biotechnol Biofuels. 2015 Sep 25;8:157. doi: 10.1186/s13068-015-0340-x (PMC4582818; doi:10.1186/s13068-015-0340-x)
Supplement: Supplementary file 1 — Additional file 1. Additional Tables, Tables S1–S4. [file 13068_2015_340_MOESM1_ESM.pdf]

ADDITIONAL TABLES

Table S1. Specific enzyme activities of CBS4435 at 25°C and BP000 at 25°C and 30°C.[a]

	CBS4435	BP000	
	25°C	25°C	30°C
CtXR[b]	0.7 ± 0.1	0.08 ± 0.01	0.10 ± 0.01
XDH	0.4 ± 0.1	0.18 ± 0.02	0.23 ± 0.03
XK	1.3 ± 0.1	0.7 ± 0.1	0.9 ± 0.1
PGI	0.23 ± 0.02	0.25 ± 0.04	0.33 ± 0.04
G6PDH	0.29 ± 0.01	0.22 ± 0.02	0.28 ± 0.02
GND	0.31 ± 0.02	0.14 ± 0.01	0.19 ± 0.02
FBA	1.0 ± 0.1	0.45 ± 0.05	0.6 ± 0.1
PFK	n.m. [c]	10 ± 1	n.d.[d]
PYK	1.2 ± 0.1	7 ± 0.05	n.d.[d]
GPP	0.030 ± 0.003	0.55 ± 0.04	n.d.[d]
ADH, NADH	1.4 ± 0.1	1.8 ± 0.3	2.5 ± 0.4
ALDH, NAD^+	n. m.[c]	n. m. [c]	n.d.[d]
$NADP^+$	n. m.[c]	0.05 ± 0.02	n.d.[d]

[a] Specific enzyme activities are shown as µmol/min/$mg_{protein}$
[b] Values refer to NADH activity
[c] not measureable
[d] not determined

Table S2. Rate equations used in kinetic modeling

Reaction	Equation
XR, R_1, R_2	$v_{tot} = v_1 + v_2$<br><br>$v_{tot} = \frac{E_t \cdot K_m^Q \left(V_f^A \left(V_r^T \left(K_{mB}^I K_i^I [A][B] + K_m^I [A][B]^2 - \frac{K_m^I [B][P][Q] + K_{mB}^I K_i^I [P][Q]}{K_{eq}^{AQ}} \right) + \frac{V_f^I K_m^T}{K_{eq}^{IT}} \left([A][B][P] - \frac{[P]^2 [Q]}{K_{eq}^{AQ}} \right) \right) + V_r^T V_f^I \left(K_{mB}^A K_i^A [I][B] + K_m^A [I][B]^2 - \frac{K_m^A [B][P][T] + K_{mB}^A K_i^A [P][T]}{K_{eq}^{IT}} + \frac{V_f^A K_m^Q}{K_{eq}^{AQ} V_r^Q} \left([I][B][P] - \frac{[P]^2 [T]}{K_{eq}^{IT}} \right) \right) \right)}{D}$<br><br>With A, NADH; I, NADPH; B, xylose; P, xylitol; Q, NAD^+; T, $NADP^+$. Superscripts AQ or IT indicate kinetic parameters determined for NAD(H) or NADP(H) dependent interconversion of xylose and xylitol<br><br>D = $V_r^T K_m^Q \Bigg(\Bigg(\left(K_{mB}^A K_m^I + K_{mB}^I K_i^I \right)[A][B] + \left(K_{mB}^I K_m^A + K_{mB}^A K_i^A \right)[I][B] + K_{mB}^I K_{mB}^A K_i^I K_i^A \left(1 + \frac{[A]}{K_i^A} + \frac{[I]}{K_i^I} + \frac{[Q]}{K_i^Q} + \frac{[T]}{K_i^T} \right) + \left(K_{mB}^A K_m^I K_i^A + K_{mB}^I K_m^A K_i^I \right)[B] + \left(\frac{V_f^I K_i^A K_m^T K_{mB}^A}{K_{eq}^{IT} V_r^T} + \frac{V_f^A K_i^I K_m^Q K_{mB}^I}{K_{eq}^{AQ} V_r^Q} \right)[P] + \left(\frac{V_f^I K_m^T K_{mB}^A}{K_{eq}^{IT} V_r^T} + \frac{V_f^A K_i^I K_{mB}^I K_m^Q}{K_{eq}^{AQ} K_i^A V_r^Q} \right)[A][P] + \left(\frac{V_f^A K_m^Q K_{mB}^I}{K_{eq}^{AQ} V_r^Q} + \frac{V_f^I K_i^A K_{mB}^A K_m^T}{K_{eq}^{IT} K_i^I V_r^T} \right)[I][P] + \left(\frac{K_{mB}^A K_i^A V_f^I}{K_{eq}^{IT} V_r^T} + \frac{K_{mB}^I K_i^I V_f^A K_m^Q}{K_{eq}^{AQ} K_i^T V_r^Q} \right)[P][T] + \left(\frac{K_{mB}^I K_i^I V_f^A}{K_{eq}^{AQ} V_r^Q} + \frac{K_{mB}^A K_i^A V_f^I K_m^T}{K_{eq}^{IT} K_i^Q V_r^T} \right)[P][Q] \Bigg)$<br>$+ K_m^A \Bigg([B]^2 \left(\frac{K_m^I [T]}{K_i^T} + [I] \left(\frac{[P]}{K_{iP}^{IT}} + 1 \right) \right) + \left(\frac{K_{mB}^A K_i^A K_m^I}{K_i^T K_m^A} + \frac{K_{mB}^I K_i^I}{K_i^T} \right)[B][T] + \left(\frac{V_f^I K_m^T}{K_i^I K_{eq}^{IT} V_r^T} + \frac{K_{mB}^A K_i^A}{K_m^A K_{iP}^{IT}} + \frac{V_f^A K_m^Q}{K_{eq}^{AQ} K_m^A V_r^Q} \right)[I][B][P] + \left(\frac{K_i^I K_{mB}^I}{K_i^T K_{mP}^T} + \frac{V_r^T K_{mB}^A K_i^A K_m^I}{V_f^I K_{iP}^{IT} K_m^A K_m^T} + \frac{V_f^A K_m^Q K_m^I}{K_{eq}^{AQ} K_i^T V_r^Q K_m^A} \right)[B][P][T] \Bigg)$<br>$+ K_m^I \Bigg([B]^2 K_m^A \left(1 + \frac{[Q]}{K_i^Q} + \frac{[A]}{K_m^A} + \frac{[A][P]}{K_{iP}^{AQ}} + \frac{V_r^T [P][T]}{K_{iP}^{IT} K_m^T V_f^I} \right) + \left(\frac{K_{mB}^I K_i^I K_m^A}{K_m^I K_i^Q} + \frac{K_{mB}^A K_i^A}{K_i^Q} \right)[B][Q] + \left(\frac{V_f^A K_m^Q}{K_{eq}^{AQ} V_r^Q} + \frac{K_m^A V_f^I K_m^T}{K_{eq}^{IT} K_m^I V_r^T} \right)[B][P] + \left(\frac{K_i^A K_{mB}^A}{K_i^Q K_{mP}^Q} + \frac{V_r^Q K_{mB}^I K_i^I K_m^A}{V_f^A K_m^I K_m^Q K_{iP}^{AQ}} + \frac{K_m^A V_f^I K_m^T}{K_{eq}^{IT} K_i^Q K_m^I V_r^T} \right)[B][P][Q] + \left(\frac{V_f^A K_m^Q}{K_i^A K_{eq}^{AQ} V_r^Q} + \frac{V_f^I K_m^T}{K_{eq}^{IT} K_m^I V_r^T} + \frac{K_{mB}^I K_i^I}{K_m^I K_{iP}^{AQ}} \right)[A][B][P] \Bigg) \Bigg)$<br>$+ \frac{V_f^I K_m^T [P]^2}{K_{eq}^{IT}} \left(\frac{V_r^Q K_m^A [B][Q]}{V_f^A K_{iP}^{AQ}} + \frac{K_m^Q [A][B]}{K_{iP}^{AQ}} + \frac{K_m^Q V_f^A [Q]}{K_{eq}^{AQ} V_r^Q} \right) + \frac{V_r^Q K_m^A K_m^I V_r^T [Q][B]^2 [P]}{V_f^A K_{iP}^{AQ}}$<br>$+ \frac{V_f^A {K_m^Q}^2 [P]^2}{K_{eq}^{AQ} V_r^Q} \left(\frac{V_r^T [I][B]}{K_{iP}^{IT}} + \frac{V_f^I}{K_{eq}^{IT}} \left([T] + K_m^T \left(1 + \frac{[I]}{K_i^I} + \frac{[A]}{K_i^A} \right) + \frac{[B][T]}{K_{iB}^{IT}} \right) \right)$

Table S2. Rate equations used in kinetic modeling (continued)

Reaction	Equation
XDH, R_3	$$v_3 = \frac{[E]V_f V_r\left([A][B]-\frac{[P][Q]}{K_{eq}}\right)}{V_r K_{iA}K_{mB}+V_r K_{mB}[A]+V_r K_{mA}[B]+\frac{V_f K_{mQ}[P]}{K_{eq}}+\frac{V_f K_{mP}[Q]}{K_{eq}}+V_r[A][B]+\frac{V_f K_{mQ}[A][P]}{K_{eq}K_{iA}}+\frac{V_f[P][Q]}{K_{eq}}+\frac{V_r K_{mA}[B][Q]}{K_{iQ}}+\frac{V_r[A][B][P]}{K_{iP}}+\frac{V_f[B][P][Q]}{K_{iB}K_{eq}}}$$ A, B, P, and Q refer to NAD^+, xylitol, xylulose, and NADH respectively while subscripts f and r indicate forward and reverse reaction.
XK, R_4<br>PP, R_5<br>UG, R_9<br>LG, R_{10}	Michaelis Menten rate equation
PGI, R_6	$$v_6 = \frac{V_f\left([F6P]-\frac{[G6P]}{K_{eq}}\right)}{[F6P]+K_{F6P}\left(1+\frac{[G6P]}{K_{G6P}}\right)}$$
G6PDH, R_7<br>GND, R_8	$$v_{7(8)} = \frac{V_{\max}[NADP^+][S]}{K_{NADP}K_{mS}\left(1+\frac{[NADPH]}{K_{iNADPH}}\right)+K_{mS}[NADP^+]+K_{NADP}[S]+[NADP^+][S]}$$ [S] corresponds to concentrations of G6P (6PG) in case of G6PDH (GND).

Table S3. Summary of kinetic modeling.[a]

Parameter	Parameter input	Exp. 1 (this study)	Exp. 2 (this study)	Exp. 3 (from [1])	Parameter input	(this study)
		BP000			CBS4435	
R^2, xylose vs. time[b]		0.9993	0.9996	0.9991		0.9997
R^2, xylitol vs. time[b]		0.998	0.993	0.988		0.9991
Biomass loading [g_{dc}/L]		0.9	3.8	1.6		1.1
Xylose [g/L]		18.0	18.0	12.0		18.0
**Reactions**	$V_{max}^{input\ c}$	$V_{max}^{pred\ c}$	$V_{max}^{pred\ c}$	$V_{max}^{pred\ c}$	$V_{max}^{input\ c}$	$V_{max}^{pred\ c}$
R_1, R_2	1.9 – 2.4	1.9 ± 0.0	2.1 ± 0.2	1.9 ± 0.0	17.8 – 21.4	19 ± 2
R_3	4.3 – 5.5	5.4 ± 0.1	5.5 ± 0.0	5.3 ±0.0	7.6 – 13.1	13.1 ± 0.0
R_4	17.0 – 21.2	21.2 ± 0.0	21.2 ± 0.1	20.20 ± 0.04	31.8 – 37.7	34 ± 3
R_5	0.01 - 10	5 ± 4	6 ± 2	5.7 ± 1.5	0.01 – 10	6 ± 4
R_6	5.8 – 8.1	7 ± 1	7 ± 1	7.2 ± 0.5	5.7 – 6.8	6.6 ± 0.2
R_7	5.7 – 7.4	6 ± 1	7 ± 1	5.9 ± 0.5	7.2 – 8.3	7.2 ± 0.0
R_8	3.6 – 4.5	3.8 ± 0.4	3.8 ± 0.4	4.0 ± 0.2	8.0 – 9.0	8.3 ± 0.4
R_9	1 – 100	3.0 ± 0.2	5.2 ± 0.7	3.4 ± 0.5	1 – 100	39 ± 1
R_{10}	1 - 100	11 ± 12	53 ± 33	20 ± 21	1 - 100	43 ± 23
**Metabolites**	$C_{i0}^{input\ d}$	$C_{i0}^{pred\ d}$	$C_{i0}^{pred\ d}$	$C_{i0}^{pred\ d}$	$C_{i0}^{input\ d}$	$C_{i0}^{pred\ d}$
Xylose	120-130 (80-85)	120.3 ± 0.6	125.6 ± 0.7	81 ± 1	120-130	124 3 ± 0.5
Xylitol	0 - 1	0.5 ± 0.1	0.98 ± 0.02	0.1 ± 0.1	0 - 1	0.28 ± 0.03
Xylulose	0	0 ± 0	0 ± 0	0 ± 0	0	0 ± 0
NADH[e]	0.2 – 0.4	0.4 ± 0.0	0.3 ± 0.1	0.3 ± 0.1	0.2 – 0.4	0.38 ± 0.02
NAD^+ [e]	0.4 – 0.6	0.5 ± 0.1	0.54 ± 0.04	0.5 ± 0.1	0.4 – 0.6	0.5 ± 0.1
NADPH[e]	0.01 – 0.03	0.012 ± 0 002	0.02 ± 0.01	0.01 ± 0.00	0.01 – 0.03	0.02 ± 0.01
$NADP^+$ [e]	0.01 – 0.04	0.02 ± 0.00	0.02 ± 0.00	0.01 ± 0.00	0.01 – 0.04	0.02 ± 0.01
C5P	0 - 1	0.6 ± 0.4	0.2 ± 0.1	0.3 ± 0.3	0 - 1	0.5 ± 0.6
F6P	0 -1	0.15 ± 0.1	0.2 ± 0.3	0.3 ± 0.4	0 -1	0.2 ± 0.3
G6P	0 - 4	1.3 ± 1.8	3.6 ± 0.3	1.6 ± 1.7	0 - 4	0.15 ± 0.10
C3P	0 - 1	0.4 ± 0.1	0.4 ± 0.4	0.3 ± 0.4	0 - 1	0.30 ± 0.25
6PG	0 - 1	0.8 ± 0.5	0.9 ± 0.1	0.6 ± 0.3	0 - 1	0.6 ± 0.2
Ethanol	0 - 1	0.2 ± 0.2	0.6 ± 0.4	0.3 ± 0.2	0 - 1	0.1 ± 0.1
CO_2	0 - 1	0.2 ± 0.4	0.45 ± 0.2	0.5 ± 0.2	0 - 1	0.6 ± 0.4

Footnotes to Table S3. Summary of kinetic modeling

[a] Predicted values shown are averages from four individual parameter estimations carried out for each experiment.

[b] Coefficient of determination R^2, calculated with $1\text{-}SS_{err}/SS_{tot}$, where SS_{err} and SS_{tot} denote the residual sum of squares and the total sum of squares, respectively.

[c] Except for R_5, R_9 and R_{10}, upper and lower limits of specific enzyme activities measured at 25°C (CBS4435) or 30°C (BP000) were used as input (V_{max}^{input}) for parameter estimation experiments. Predicted values are indicated by a superscript ^{pred}. A protein content in the dry biomass of 40% was assumed [2]. V_{max} values are shown as mmol/g_{dc}/h.

[d] C_{i0}, shown as mM, referred to initial concentrations applied to (superscript ^{input}) and predicted (superscript ^{pred}) by the kinetic model. Metabolite concentrations were not fixed during time course simulations.

[e] Concentration ranges reported previously for BP000 metabolizing xylose under anaerobic conditions were applied [3]. A specific cell volume of 2.38 mL per g_{dc} was used [4].

Table S4. Reactant concentrations in enzyme activity assays

Enzyme	Concentrations in assay
Xylose reductase (XR)	
PPB [a], pH 7.0	100 mM
Xylose	672 mM
NADH	250 µM
Xylitol dehydrogenase (XDH)	
PPB [a], pH 7.0	100 mM
Xylitol	144 mM
NAD^+	3 mM
Xylulokinase (XK)	
HEPES, pH 7.0,	44 mM
NADH	0.2 mM
Xylulose	4.3 mM
ATP	5 mM
Phosphoenolpyruvate	1 mM
$MgCL_2$ $6H_2O$	8.8 mM
BSA	0.88 g L^{-1}
Pyruvate kinase / lactate dehydrogenase enzymes from rabbit muscle	> 3.6 / 2.4 µmol/min/mL [b]
Phosphoglucose isomerase (PGI)	
PPB [a], pH 7.0	100 mM
Glucose 6-phosphate	20 mM
Mannitol-1-phosphate 5-dehydrogenase from *Aspergillus fumigatus* [c]	4.0 µmol/min/mL
NADH	200 µM
Alcohol dehydrogenase (ADH)	
PPB [a], pH 7.0	100 mM
Acetaldehyde	4.8 mM
NADH	200 µM
Acetaldehyde dehydrogenase (ALDH)	
PPB [a], pH 7.0	100 mM
Acetaldehyde	4.8 mM
$NAD(P)^+$	2 mM
Glucose 6-phosphate dehydrogenase (G6PDH)	
PPB [a], pH 7.0	100 mM
Glucose 6-phosphate	19 mM
$NADP^+$	2 mM
6-Phosphogluconate dehydrogenase (GND)	
PPB [a], pH 7.0	100 mM
6-Phosphogluconate	1 mM
$NADP^+$	2 mM

Table S4. Reactant concentrations in enzyme activity assays (continued)

Enzyme	Concentrations in assay
Aldolase (FBA)	
PPB [a], pH 7.4	100 mM
Fructose 1,6-bisphosphate	1 mM
$MgCl_2$ $6H_2O$	10 mM
NADH	0.2 mM
Triosephosphate isomerase / α-glycerol-3P dehydrogenase from rabbit muscle	> 1 / 10 µmol/min/mL[b]
6-Phosphofructokinase (PFK)	
PPB [a], pH 7.4	100 mM
Fructose-6P	5 mM
ATP	1 mM
$MgCl_2$ $6H_2O$	10 mM
NADH	0.2 mM
Aldolase	> 2 µmol/min/mL[b]
Triosephosphate isomerase / α-glycerol-3P dehydrogenase from rabbit muscle	> 1 / 10 µmol/min/mL[b]
Pyruvate kinase (PYK)	
PPB[a], pH 7.0	100 mM
Phosphoenolpyruvate	3.0 mM
ADP	1.5 mM
FBP	1.5 mM
NADH	0.2 mM
Lactate dehydrogenase from rabbit muscle	> 2 µmol/min/mL[b]
Glycerol 3-phosphate phosphatase (GPP)	
Tris/HCl, pH 7.5	50 mM
$MgCl_2$	2.0 mM
Glycerol 3-phosphate	10 mM

[a] PPB, potassium phosphate buffer

[b] According to manufacturer's information (Sigma-Aldrich, Missouri, U.S.A.)

[c] Pure fractions with a specific activity of 235 µmol/min/mg of mannitol-1-phosphate 5-dehydrogenase were prepared as described by [5].

References

1. Petschacher B, Nidetzky B. Altering the coenzyme preference of xylose reductase to favor utilization of NADH enhances ethanol yield from xylose in a metabolically engineered strain of *Saccharomyces cerevisiae*. Microbial cell factories. 2008;7:9.
2. Lange HC, Heijnen JJ. Statistical reconciliation of the elemental and molecular biomass composition of *Saccharomyces cerevisiae*. Biotechnology and bioengineering. 2001;75(3):334-44.
3. Klimacek M, Krahulec S, Sauer U, Nidetzky B. Limitations in xylose-fermenting *Saccharomyces cerevisiae*, made evident through comprehensive metabolite profiling and thermodynamic analysis. Applied and environmental microbiology. 2010;76(22):7566-74. doi:10.1128/AEM.01787-10.
4. Ditzelmüller G, Wöhrer W, Kubicek CP, Röhr M. Nucleotide pools of growing, synchronized and stressed cultures of *Saccharomyces cerevisiae*. Arch Microbiol. 1983;135(1):63-7.
5. Krahulec S, Armao GC, Weber H, Klimacek M, Nidetzky B. Characterization of recombinant *Aspergillus fumigatus* mannitol-1-phosphate 5-dehydrogenase and its application for the stereoselective synthesis of protio and deuterio forms of D-mannitol 1-phosphate. Carbohydr Res. 2008;343(9):1414-23. doi:10.1016/j.carres.2008.04.011.
