# Supplementary material for: Identification of novel metabolic interactions controlling carbon flux from xylose to ethanol in natural and recombinant yeasts
Source: Biotechnol Biofuels. 2015 Sep 25;8:157. doi: 10.1186/s13068-015-0340-x (PMC4582818; doi:10.1186/s13068-015-0340-x)
Supplement: Supplementary file 2 — Additional file 2. Additional Schemes and Figures; Scheme S1, Figures S1–S3. [file 13068_2015_340_MOESM2_ESM.pdf]

ADDITIONAL SCHEMES AND FIGURES

Scheme S1

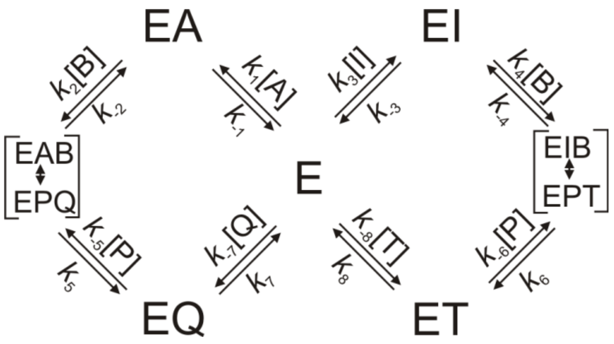

Scheme S1. Reversible ordered Bi Bi steady-state reaction mechanism accounting for alternate binding of the first substrate. E, B, P, A, Q, I, and T referred to free enzyme, xylose, xylitol, NADH, NAD^+, NADPH, and $NADP^+$, respectively. EA, EQ, EI and ET represent binary and EAB, EPQ, EIB and EPT represent ternary enzyme-reactant complexes. Microscopic rate constants are indicated. Interconversion at the ternary complex level is assumed to be fast.

Figure S1

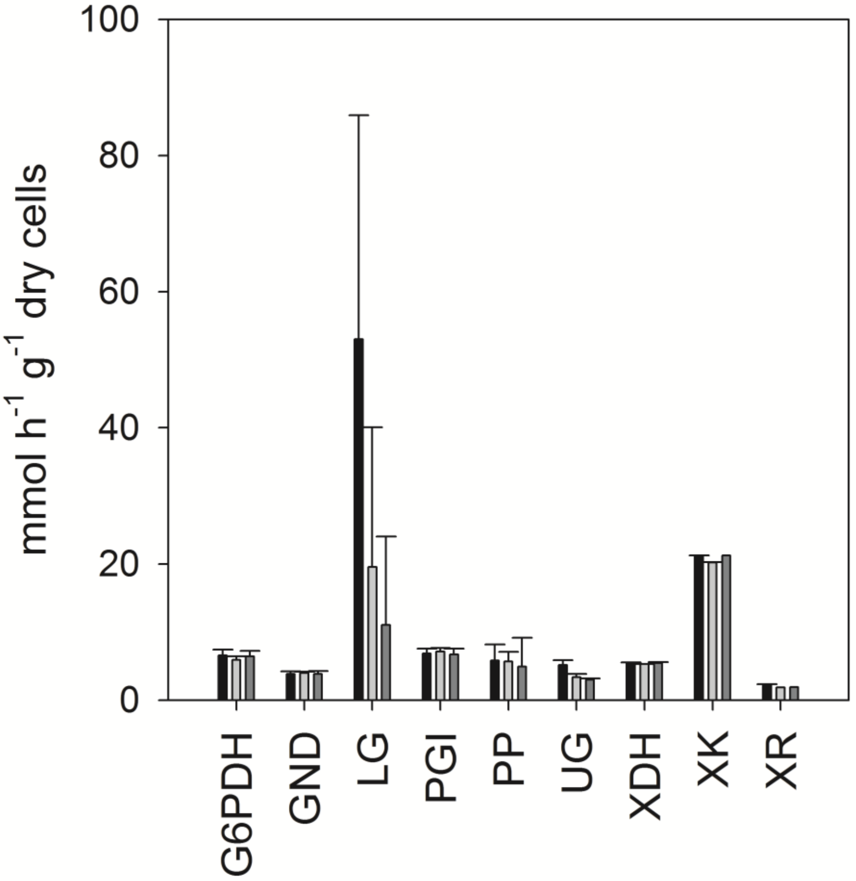

Figure S1. Summary of V_{max} values obtained from parameter estimation analysis for BP000 xylose fermentations at different initial xylose and biomass concentrations. Initial conditions were as follows: Black bars: 3.8 g_{dc}/L, 18 g/L xylose (Exp. 2, this study), light grey bars: 1.6 g_{dc}/L, 12 g/L xylose (Exp. 3, [1]); dark grey bars: 0.9 g_{dc}/L, 18 g/L xylose (Exp. 1, this study). Experiments relate to conditions described in Additional File 1, Table S3. Error bars indicate variation obtained from 4 individual parameter estimation experiments.

Figure S2

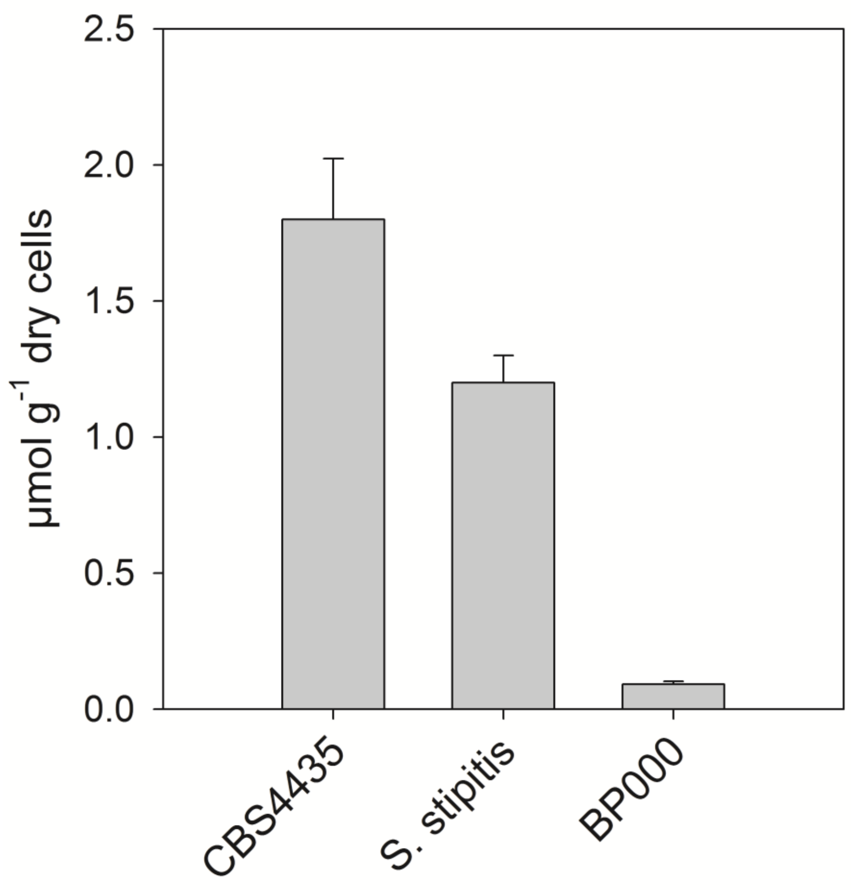

Figure S2. Intracellular concentrations of Glyc3P determined for BP000, CBS4435 and *S. stipitis*. Data for *S. stipitis* was obtained at the pseudo steady-state of anaerobic xylose fermentation as described for BP000 and CBS4435 in the main text.

Figure S3

NADP+	NAD+	NADPH	NADH
0	0	0.1	0
0	0	0	0.1
0	0	0.1	0.1
0.02	0	0.1	0
0.02	0	0	0.1
0.02	0	0.1	0.1
0	0.4	0.1	0
0	0.4	0	0.1
0	0.4	0.1	0.1
0.02	0.4	0.1	0
0.02	0.4	0	0.1
0.02	0.4	0.1	0.1
0.01	0	0.1	0.1
0.1	0	0.1	0.1
0.4	0	0.1	0.1
0	0.01	0.1	0.1
0	0.1	0.1	0.1
0	0.4	0.1	0.1

Xylose: 133 mM, pH 7.0, 30°C

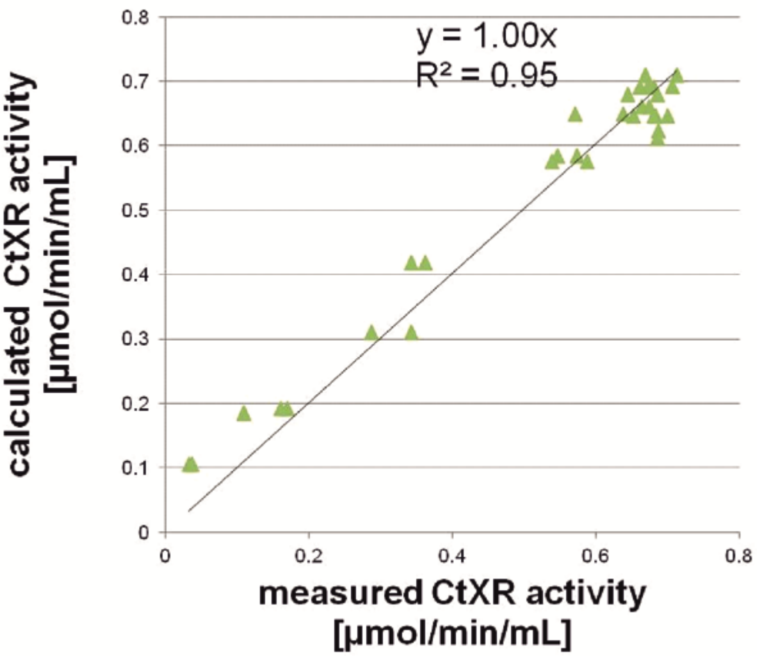

Figure S3. Experimental verification of the rate equation derived for CtXR. Table on the left hand side shows sets of reactant concentrations applied (in mM).

References

1. Petschacher B, Nidetzky B. Altering the coenzyme preference of xylose reductase to favor utilization of NADH enhances ethanol yield from xylose in a metabolically engineered strain of *Saccharomyces cerevisiae*. Microbial cell factories. 2008;7:9.
