# Supplementary material for: Identification of novel metabolic interactions controlling carbon flux from xylose to ethanol in natural and recombinant yeasts
Source: Biotechnol Biofuels. 2015 Sep 25;8:157. doi: 10.1186/s13068-015-0340-x (PMC4582818; doi:10.1186/s13068-015-0340-x)
Supplement: Supplementary file 3 — Additional file 3. Additional information; preparation of intracellular metabolites; derivation of the complete rate law describing simultaneous utilization of NAD(P)H by XR; validation of XR rate equation; acquisition of model-relevant kinetic data; activity of CtXR with DHAP. [file 13068_2015_340_MOESM3_ESM.pdf]

ADDITIONAL INFORMATION

Preparation of intracellular metabolite extracts

The volume ratio of sample to quenching solution (100% methanol was used) was 1:20. The quenching solution was precooled on dry ice. Cell-methanol suspensions were centrifuged for 3 min at 5000 rpm and -9°C. Quenched cells were stored at -80°C (no longer than 1 week prior analysis). Prior to metabolite extraction with boiling ethanol 50 µL of a ^{13}C- *S. cerevisiae* CEN-PK 113-7D metabolite extract (ISTD), precooled on ice, prepared as described by [1], was added for metabolite-specific internal standardization. Metabolite extracts were dried under a constant nitrogen (5.0) flow using an Evaporator® (Liebisch Labortechnik, Germany) and resolved in 100 µL LC/MS-grade water (Sigma-Aldrich, Missouri, U.S.A.). Remaining solids were removed by centrifugation (13200 rpm, 20°C, 10 min).

Derivation of the complete rate law describing simultaneous utilization of NAD(P)H by XR

The complete rate equation describing simultaneous usage of NAD(P)(H) in the reversible interconversion of xylose and xylitol by CtXR was not known and therefore derived from scratch by principles first developed by King-Altman [2]. The reaction mechanism of CtXR is depicted in Additional File 2, Scheme S1. The King-Altman software tool provided by the open source Biokin.COM (http://www.biokin.com/king-altman/index.html) was used to generate, based on the overall reaction shown below, numerator (*N*) and denominator (*D*) terms and coefficients of the general velocity equation of the form $v = N/D$. After multiplying *N* and *D* coefficients by a common factor term coefficients were substituted appropriately by expressions, formed by microscopic rate constants, which define steady-state kinetic parameters measurable by standard *in vitro* assays. Symbolic algebra was carried out with Matlab R2007b (Mathworks, Inc., U.S.A.) while substitutions were done manually after definition of substitution rules.

Overall reaction

A + I + B = P + Q + T

With A, NADH; I, NADPH; B, xylose; P, xylitol; Q, NAD^+; T, $NADP^+$

Equations

k_{+1}: A + E → EA
k_{-1}: EA → A + E
k_{+2}: B + EA → EXY
k_{-2}: EXY → B + EA
k_{+3}: I + E → EI
k_{-3}: EI → I + E
k_{+4}: B + EI → VXY
k_{-4}: VXY → B + EI

k_{+5}: EXY → P + EQ
k_{-5}: P + EQ → EXY
k_{+6}: VXY → P + ET
k_{-6}: P + ET → VXY
k_{+7}: EQ → Q + E
k_{-7}: Q + E → EQ
k_{+8}: ET → T + E
k_{-8}: T + E → ET

A, I, B, Q, T were as defined as above; E, free enzyme; binary enzyme-coenzyme complexes: EA, E-NADH; EI, E-NADPH; EQ, E-NAD^+; ET, E-$NADP^+$; ternary enzyme-coenzyme-substrate complexes: EXY, E-NADH-xylose; VXY, E-NADPH-xylose

General rate equation

$v = \boldsymbol{N}/\boldsymbol{D} = \mathrm{d[P]/d}t = + k_{+5}\,[\mathrm{EXY}]' - k_{-5}\,[\mathrm{P}]\,[\mathrm{EQ}]' + k_{+6}\,[\mathrm{VXY}]' - k_{-6}\,[\mathrm{P}]\,[\mathrm{ET}]'$

Numerator:

$\boldsymbol{N} = n_1\,[\mathrm{P}]^2[\mathrm{T}] + n_2\,[\mathrm{P}]^2[\mathrm{Q}] + n_3\,[\mathrm{B}][\mathrm{P}][\mathrm{T}] + n_4\,[\mathrm{B}][\mathrm{P}][\mathrm{Q}] + n_5\,[\mathrm{I}][\mathrm{B}][\mathrm{P}] + n_6\,[\mathrm{I}][\mathrm{B}]^2 + n_7\,[\mathrm{A}][\mathrm{B}][\mathrm{P}] + n_8\,[\mathrm{A}][\mathrm{B}]^2 + n_9\,[\mathrm{P}][\mathrm{T}] + n_{10}\,[\mathrm{P}][\mathrm{Q}] + n_{11}\,[\mathrm{I}][\mathrm{B}] + n_{12}\,[\mathrm{A}][\mathrm{B}]$

Numerator coefficients were defined by

$n_1 = - k_{-1}\,k_{-2}\,k_{-3}\,k_{-4}\,k_{-5}\,k_{-6}\,k_{-8}$
$n_2 = - k_{-1}\,k_{-2}\,k_{-3}\,k_{-4}\,k_{-5}\,k_{-6}\,k_{-7}$
$n_3 = - k_2\,k_{-3}\,k_{-4}\,k_5\,k_{-6}\,k_7\,k_{-8}$
$n_4 = - k_{-1}\,k_{-2}\,k_4\,k_{-5}\,k_6\,k_{-7}\,k_8$
$n_5 = + k_{-1}\,k_{-2}\,k_3\,k_4\,k_{-5}\,k_6\,k_8$
$n_6 = + k_2\,k_3\,k_4\,k_5\,k_6\,k_7\,k_8$
$n_7 = + k_1\,k_2\,k_{-3}\,k_{-4}\,k_5\,k_{-6}\,k_7$
$n_8 = + k_1\,k_2\,k_4\,k_5\,k_6\,k_7\,k_8$
$n_9 = - k_{-1}\,k_{-2}\,k_{-3}\,k_{-4}\,k_{-6}\,k_7\,k_{-8} - k_{-1}\,k_{-3}\,k_{-4}\,k_5\,k_{-6}\,k_7\,k_{-8}$
$n_{10} = - k_{-1}\,k_{-2}\,k_{-3}\,k_{-4}\,k_{-5}\,k_{-7}\,k_8 - k_{-1}\,k_{-2}\,k_{-3}\,k_{-5}\,k_6\,k_{-7}\,k_8$
$n_{11} = + k_{-1}\,k_{-2}\,k_3\,k_4\,k_6\,k_7\,k_8 + k_{-1}\,k_3\,k_4\,k_5\,k_6\,k_7\,k_8$
$n_{12} = + k_1\,k_2\,k_{-3}\,k_{-4}\,k_5\,k_7\,k_8 + k_1\,k_2\,k_{-3}\,k_5\,k_6\,k_7\,k_8$

Denominator:

$\boldsymbol{D} = d_1\,[\mathrm{B}][\mathrm{P}]^2[\mathrm{T}] + d_2\,[\mathrm{B}][\mathrm{P}]^2[\mathrm{Q}] + d_3\,[\mathrm{B}]^2[\mathrm{P}][\mathrm{T}] + d_4\,[\mathrm{B}]^2[\mathrm{P}][\mathrm{Q}] + d_5\,[\mathrm{I}][\mathrm{B}][\mathrm{P}]^2 + d_6\,[\mathrm{I}][\mathrm{B}]^2[\mathrm{P}] + d_7\,[\mathrm{A}][\mathrm{B}][\mathrm{P}]^2 + d_8\,[\mathrm{A}][\mathrm{B}]^2[\mathrm{P}] + d_9\,[\mathrm{P}]^2[\mathrm{T}] + d_{10}\,[\mathrm{P}]^2[\mathrm{Q}] + d_{11}\,[\mathrm{B}][\mathrm{P}][\mathrm{T}] + d_{12}\,[\mathrm{B}][\mathrm{P}][\mathrm{Q}] + d_{13}\,[\mathrm{B}]^2[\mathrm{T}] + d_{14}\,[\mathrm{B}]^2[\mathrm{Q}] + d_{15}\,[\mathrm{I}][\mathrm{P}]^2 + d_{16}\,[\mathrm{I}][\mathrm{B}][\mathrm{P}] + d_{17}\,[\mathrm{I}][\mathrm{B}]^2 + d_{18}\,[\mathrm{A}][\mathrm{P}]^2 + d_{19}\,[\mathrm{A}][\mathrm{B}][\mathrm{P}] + d_{20}\,[\mathrm{A}][\mathrm{B}]^2 + d_{21}\,[\mathrm{P}][\mathrm{T}] + d_{22}\,[\mathrm{P}][\mathrm{Q}] + d_{23}\,[\mathrm{P}]^2 + d_{24}\,[\mathrm{B}][\mathrm{T}] + d_{25}\,[\mathrm{B}][\mathrm{Q}] +$

d_{26} [B][P] + d_{27} $[B]^2$ + d_{28} [I][P] + d_{29} [I][B] + d_{30} [A][P] + d_{31} [A][B] + d_{32} [T] + d_{33} [Q] +

d_{34} [P] + d_{35} [B] + d_{36} [I] + d_{37} [A] + d_{38}

Denominator coefficients were defined by

$d_1 = k_{-1} k_{-2} k_4 k_{-5} k_{-6} k_{-8}$

$d_2 = k_2 k_{-3} k_{-4} k_{-5} k_{-6} k_{-7}$

$d_3 = k_2 k_4 k_5 k_{-6} k_7 k_{-8}$

$d_4 = k_2 k_4 k_{-5} k_6 k_{-7} k_8$

$d_5 = k_{-1} k_{-2} k_3 k_4 k_{-5} k_{-6}$

$d_6 = k_2 k_3 k_4 k_5 k_{-6} k_7$

$d_7 = k_1 k_2 k_{-3} k_{-4} k_{-5} k_{-6}$

$d_8 = k_1 k_2 k_4 k_{-5} k_6 k_8$

$d_9 = k_{-1} k_{-2} k_{-4} k_{-5} k_{-6} k_{-8} + k_{-1} k_{-2} k_{-3} k_{-5} k_{-6} k_{-8}$

$d_{10} = k_{-2} k_{-3} k_{-4} k_{-5} k_{-6} k_{-7} + k_{-1} k_{-3} k_{-4} k_{-5} k_{-6} k_{-7}$

$d_{11} = k_2 k_{-4} k_5 k_{-6} k_7 k_{-8} + k_{-1} k_{-2} k_4 k_{-6} k_7 k_{-8} + k_{-1} k_4 k_5 k_{-6} k_7 k_{-8} + k_2 k_{-3} k_5 k_{-6} k_7 k_{-8}$
$+ k_{-1} k_{-2} k_4 k_{-5} k_6 k_{-8}$

$d_{12} = k_{-2} k_4 k_{-5} k_6 k_{-7} k_8 + k_{-1} k_4 k_{-5} k_6 k_{-7} k_8 + k_2 k_{-3} k_{-4} k_{-5} k_{-7} k_8 + k_2 k_{-3} k_{-5} k_6 k_{-7} k_8$
$+ k_2 k_{-3} k_{-4} k_5 k_{-6} k_{-7}$

$d_{13} = k_2 k4 k_5 k_6 k_7 k_{-8}$

$d_{14} = k_2 k_4 k_5 k_6 k_{-7} k_8$

$d_{15} = k_{-1} k_{-2} k_3 k_{-4} k_{-5} k_{-6}$

$d_{16} = k_2 k_3 k_{-4} k_5 k_{-6} k_7 + k_{-1} k_{-2} k_3 k_4 k_{-6} k_7 + k_{-1} k_{-2} k_3 k_4 k_{-5} k_8 + k_{-1} k_3 k_4 k_5 k_{-6} k_7$
$+ k_{-1} k_{-2} k_3 k_4 k_{-5} k_6$

$d_{17} = k_2 k_3 k_4 k_5 k_7 k_8 + k_2 k_3 k_4 k_5 k_6 k_7$

$d_{18} = k_1 k_{-2} k_{-3} k_{-4} k_{-5} k_{-6}$

$d_{19} = k_1 k_{-2} k_4 k_{-5} k_6 k_8 + k_1 k_2 k_{-3} k_{-4} k_{-6} k_7 + k_1 k_2 k_{-3} k_{-4} k_{-5} k_8 + k_1 k_2 k_{-3} k_{-5} k_6 k_8$
$+ k_1 k_2 k_{-3} k_{-4} k_5 k_{-6}$

$d_{20} = k_1 k_2 k_4 k_6 k_7 k_8 + k_1 k_2 k_4 k_5 k_6 k_8$

$d_{21} = k_{-1} k_{-2} k_{-4} k_{-6} k_7 k_{-8} + k_{-1} k_{-4} k_5 k_{-6} k_7 k_{-8} + k_{-1} k_{-2} k_{-3} k_{-6} k_7 k_{-8} + k_{-1} k_{-3} k_5 k_{-6} k_7 k_{-8}$
$+ k_{-1} k_{-2} k_{-3} k_{-4} k_{-5} k_{-8} + k_{-1} k_{-2} k_{-3} k_{-5} k_6 k_{-8}$

$d_{22} = k_{-2} k_{-3} k_{-4} k_{-5} k_{-7} k_8 + k_{-2} k_{-3} k_{-5} k_6 k_{-7} k_8 + k_{-1} k_{-3} k_{-4} k_{-5} k_{-7} k_8 + k_{-1} k_{-3} k_{-5} k_6 k_{-7} k_8$
$+ k_{-1} k_{-2} k_{-3} k_{-4} k_{-6} k_{-7} + k_{-1} k_{-3} k_{-4} k_5 k_{-6} k_{-7}$

$d_{23} = k_{-1} k_{-2} k_{-3} k_{-4} k_{-5} k_{-6}$

$d_{24} = k_{-1} k_{-2} k_4 k_6 k_7 k_{-8} + k_{-1} k_4 k_5 k_6 k_7 k_{-8} + k_2 k_{-3} k_{-4} k_5 k_7 k_{-8} + k_2 k_{-3} k_5 k_6 k_7 k_{-8}$

$d_{25} = k_{-1} k_{-2} k_4 k_6 k_{-7} k_8 + k_{-1} k_4 k_5 k_6 k_{-7} k_8 + k_2 k_{-3} k_{-4} k_5 k_{-7} k_8 + k_2 k_{-3} k_5 k_6 k_{-7} k_8$

$d_{26} = k_{-1} k_{-2} k_4 k_{-5} k_6 k_8 + k_2 k_{-3} k_{-4} k_5 k_{-6} k_7$

$d_{27} = k_2 k_4 k_5 k_6 k_7 k_8$

$d_{28} = k_{-1} k_{-2} k_3 k_{-4} k_{-6} k_7 + k_{-1} k_{-2} k_3 k_{-4} k_{-5} k_8 + k_{-1} k_{-2} k_3 k_{-5} k_6 k_8 + k_{-1} k_3 k_{-4} k_5 k_{-6} k_7$

$d_{29} = k_2 k_3 k_{-4} k_5 k_7 k_8 + k_2 k_3 k_5 k_6 k_7 k_8 + k_{-1} k_{-2} k_3 k_4 k_7 k_8 + k_{-1} k_3 k_4 k_5 k_7 k_8$
$+ k_{-1} k_{-2} k_3 k_4 k_6 k_7 + k_{-1} k_3 k_4 k_5 k_6 k_7$

$d_{30} = k_1 k_{-2} k_{-3} k_{-4} k_{-6} k_7 + k_1 k_{-2} k_{-3} k_{-4} k_{-5} k_8 + k_1 k_{-2} k_{-3} k_{-5} k_6 k_8 + k_1 k_{-3} k_{-4} k_5 k_{-6} k_7$

$d_{31} = k_1 k_{-2} k_4 k_6 k_7 k_8 + k_1 k_4 k_5 k_6 k_7 k_8 + k_1 k_2 k_{-3} k_{-4} k_7 k_8 + k_1 k_2 k_{-3} k_6 k_7 k_8$
$+ k_1 k_2 k_{-3} k_{-4} k_5 k_8 + k_1 k_2 k_{-3} k_5 k_6 k_8$

$d_{32} = k_{-1} k_{-2} k_{-3} k_{-4} k_7 k_{-8} + k_{-1} k_{-2} k_{-3} k_6 k_7 k_{-8} + k_{-1} k_{-3} k_{-4} k_5 k_7 k_{-8} + k_{-1} k_{-3} k_5 k_6 k_7 k_{-8}$

$d_{33} = k_{-1} k_{-2} k_{-3} k_{-4} k_{-7} k_8 + k_{-1} k_{-2} k_{-3} k_6 k_{-7} k_8 + k_{-1} k_{-3} k_{-4} k_5 k_{-7} k_8 + k_{-1} k_{-3} k_5 k_6 k_{-7} k_8$

$d_{34} = k_{-1} k_{-2} k_{-3} k_{-4} k_{-6} k_7 + k_{-1} k_{-2} k_{-3} k_{-4} k_{-5} k_8 + k_{-1} k_{-2} k_{-3} k_{-5} k_6 k_8 + k_{-1} k_{-3} k_{-4} k_5 k_{-6} k_7$

$d_{35} = k_{-1} k_{-2} k_4 k_6 k_7 k_8 + k_{-1} k_4 k_5 k_6 k_7 k_8 + k_2 k_{-3} k_{-4} k_5 k_7 k_8 + k_2 k_{-3} k_5 k_6 k_7 k_8$

$d_{36} = k_{-1}\, k_{-2}\, k_3\, k_{-4}\, k_7\, k_8 + k_{-1}\, k_{-2}\, k_3\, k_6\, k_7\, k_8 + k_{-1}\, k_3\, k_{-4}\, k_5\, k_7\, k_8 + k_{-1}\, k_3\, k_5\, k_6\, k_7\, k_8$
$d_{37} = k_1\, k_{-2}\, k_{-3}\, k_{-4}\, k_7\, k_8 + k_1\, k_{-2}\, k_{-3}\, k_6\, k_7\, k_8 + k_1\, k_{-3}\, k_{-4}\, k_5\, k_7\, k_8 + k_1\, k_{-3}\, k_5\, k_6\, k_7\, k_8$
$d_{38} = k_{-1}\, k_{-2}\, k_{-3}\, k_{-4}\, k_7\, k_8 + k_{-1}\, k_{-2}\, k_{-3}\, k_6\, k_7\, k_8 + k_{-1}\, k_{-3}\, k_{-4}\, k_5\, k_7\, k_8 + k_{-1}\, k_{-3}\, k_5\, k_6\, k_7\, k_8$

Rules and definitions

Factor for multiplying numerator and denominator terms

$$\frac{coefB^2 coefP^2 numP^2T}{coefAB^2 coefIB^2 coefP^2T coefP^2Q}$$

Definitions

$$V_{\mathrm{f}}^{A} = \frac{numAB^2}{coefAB^2};\ V_{\mathrm{f}}^{I} = \frac{numIB^2}{coefIB^2};\ V_{\mathrm{r}}^{Q} = \frac{numP^2Q}{coefP^2Q};\ V_{\mathrm{r}}^{T} = \frac{numP^2T}{coefP^2T}$$

$$K_{\mathrm{i}}^{A} = \frac{const}{coefA};\ K_{\mathrm{i}}^{I} = \frac{const}{coefI};\ K_{\mathrm{i}}^{Q} = \frac{const}{coefQ};\ K_{\mathrm{i}}^{T} = \frac{const}{coefT}$$

$$K_{\mathrm{m}}^{I} = \frac{coefB^2}{coefIB^2};\ K_{\mathrm{m}}^{A} = \frac{coefB^2}{coefAB^2};\ K_{\mathrm{m}}^{Q} = \frac{coefP^2}{coefP^2Q};\ K_{\mathrm{m}}^{T} = \frac{coefP^2}{coefP^2T}$$

$$K_{\mathrm{mB}}^{A} = \frac{numIB\ coefB^2\ coefA}{coefAB^2\ const\ numIB^2};\ K_{\mathrm{mB}}^{I} = \frac{numAB\ coefB^2\ coefI}{coefIB^2\ const\ numAB^2};\ K_{\mathrm{mP}}^{Q} = \frac{numPQ\ coefP^2\ coefQ}{coefP^2Q\ const\ numP^2T};$$
$$K_{\mathrm{mP}}^{T} = \frac{numPT\ coefP^2\ coefT}{coefP^2T\ const\ numP^2Q}$$

Maximum rate constants, V_{f} and V_{r}

$$V_{\mathrm{f}}^{A} = \frac{k_5k_7}{k_5+k_7};\ V_{\mathrm{f}}^{I} = \frac{k_6k_8}{k_6+k_8};\ V_{\mathrm{r}}^{Q} = \frac{k_{-1}k_{-2}}{k_{-1}+k_{-2}};\ V_{\mathrm{r}}^{T} = \frac{k_{-3}k_{-4}}{k_{-3}+k_{-4}}$$

Coenzyme dissociation constants, K_{i}

$$K_{\mathrm{i}}^{A} = \frac{k_{-1}}{k_1};\ K_{\mathrm{i}}^{I} = \frac{k_{-3}}{k_3};\ K_{\mathrm{i}}^{Q} = \frac{k_7}{k_{-7}};\ K_{\mathrm{i}}^{T} = \frac{k_8}{K_{-8}}$$

Michaelis constants, K_{m}, K_{mB}, and K_{mP}

$$K_{\mathrm{m}}^{A} = \frac{k_5k_7}{k_1(k_5+k_7)};\ K_{\mathrm{m}}^{I} = \frac{k_6k_8}{k_3(k_6+k_8)};\ K_{\mathrm{m}}^{Q} = \frac{k_{-1}k_{-2}}{k_{-7}(k_{-1}+k_{-2})};\ K_{\mathrm{m}}^{T} = \frac{k_{-3}k_{-4}}{k_{-8}(k_{-3}+k_{-4})}$$

$$K_{\mathrm{mB}}^{A} = \frac{k_7(k_{-2}+k_5)}{k_2(k_5+k_7)}\,;\ K_{\mathrm{mB}}^{I} = \frac{k_8(k_{-4}+k_6)}{k_4(k_8+k_6)};\ K_{\mathrm{mP}}^{Q} = \frac{k_{-1}(k_{-2}+k_5)}{k_{-5}(k_{-1}+k_{-2})};\ K_{\mathrm{mP}}^{T} = \frac{k_{-3}(k_{-4}+k_6)}{k_{-6}(k_{-3}+k_{-4})}$$

Substrate and product dissociation constants, K_{iB} and K_{iP}

$$K_{\mathrm{iB}}^{AQ} = \frac{k_{-1}+k_{-2}}{k_2};\ K_{\mathrm{iB}}^{IT} = \frac{k_{-3}+k_{-4}}{k_4};\ K_{\mathrm{iP}}^{AQ} = \frac{k_5+k_7}{k_{-5}};\ K_{\mathrm{iP}}^{IT} = \frac{k_6+k_8}{k_{-6}}$$

Substrate and product dissociation constants from the central complexes, K_{B} and K_{P}

$$K_{\mathrm{B}}^{A} = \frac{k_{-2}}{k_2};\ K_{\mathrm{B}}^{I} = \frac{k_{-4}}{k_4};\ K_{\mathrm{P}}^{Q} = \frac{k_5}{k_{-5}};\ K_{\mathrm{P}}^{T} = \frac{k_6}{k_{-6}}$$

Equilibrium constants, K_{eq}

$$K_{\mathrm{eq}}^{AQ} = \frac{k_1k_2k_5k_7}{k_{-1}k_{-2}k_{-5}k_{-7}};\ K_{\mathrm{eq}}^{IT} = \frac{k_3k_4k_6k_8}{k_{-3}k_{-4}k_{-6}k_{-8}}$$

Relationships used for simplification

$$K_{\mathrm{eq}} = \frac{V_{\mathrm{f}}K_{\mathrm{iQ}}K_{\mathrm{mP}}}{V_{\mathrm{r}}K_{\mathrm{iA}}K_{\mathrm{mB}}};\ K_{\mathrm{eq}} = \left(\frac{V_f}{V_r}\right)^2 \frac{K_{\mathrm{iP}}K_{\mathrm{mQ}}}{K_{\mathrm{iB}}K_{\mathrm{mA}}}$$

$$K_{\mathrm{B}} = \frac{K_{\mathrm{iB}}K_{\mathrm{mA}}V_{\mathrm{r}}}{K_{\mathrm{iA}}V_{\mathrm{f}}};\ K_{\mathrm{P}} = \frac{K_{\mathrm{iP}}K_{\mathrm{mQ}}V_{\mathrm{f}}}{K_{\mathrm{iQ}}V_{\mathrm{r}}}$$

Superscripts AQ or IT indicate kinetic parameters determined for NAD(H) or NADP(H) dependent interconversion of xylose and xylitol.

Expressions obtained for numerator coefficients (*num*)

1. *num*P^2T = - $(K_{\mathrm{m}}^{Q})^2 * V_{\mathrm{f}}^{I} * V_{\mathrm{f}}^{A} * V_{\mathrm{r}}^{T} * [P]^2*[T] / (K_{\mathrm{eq}}^{IT} * K_{\mathrm{eq}}^{AQ} * V_{\mathrm{r}}^{Q})$
2. *num*P^2Q = - $V_{\mathrm{f}}^{I} * V_{\mathrm{f}}^{A} * K_{\mathrm{m}}^{Q} * K_{\mathrm{m}}^{T} * [P]^2*[Q] / (K_{\mathrm{eq}}^{IT} * K_{\mathrm{eq}}^{AQ})$
3. *num*BPT = - $K_{\mathrm{m}}^{Q} * V_{\mathrm{r}}^{T} * K_{\mathrm{m}}^{A} * V_{\mathrm{f}}^{I} * [B]*[P]*[T] / K_{\mathrm{eq}}^{IT}$
4. *num*BPQ = - $K_{\mathrm{m}}^{Q} * V_{\mathrm{r}}^{T} * K_{\mathrm{m}}^{I} * V_{\mathrm{f}}^{A} * [B]*[P]*[Q] / K_{\mathrm{eq}}^{AQ}$
5. *num*IBP = + $(K_{\mathrm{m}}^{Q})^2 * V_{\mathrm{f}}^{I} * V_{\mathrm{f}}^{A} * V_{\mathrm{r}}^{T}\ [I]*[B]*[P] / (\mathrm{K}_{\mathrm{eq}}^{AQ} * \mathrm{V}_{\mathrm{r}}^{Q})$
6. *num*IB^2 = + $K_{\mathrm{m}}^{Q} * V_{\mathrm{r}}^{T} * K_{\mathrm{m}}^{A} * V_{\mathrm{f}}^{I} * [I]*[B]^2$
7. *num*ABP = + $K_{\mathrm{m}}^{Q} * V_{\mathrm{f}}^{I} * V_{\mathrm{f}}^{A} * K_{\mathrm{m}}^{T} * [A]*[B]*[P] / K_{\mathrm{eq}}^{IT}$
8. *num*AB^2 = + $V_{\mathrm{r}}^{T} * K_{\mathrm{m}}^{Q} * V_{\mathrm{f}}^{A} * K_{\mathrm{m}}^{I} * [A]*[B]^2$
9. *num*PT = - $K_{\mathrm{m}}^{Q} * V_{\mathrm{r}}^{T} * V_{\mathrm{f}}^{I} * K_{\mathrm{mB}}^{A} * K_{\mathrm{i}}^{A} * [P]*[T] / K_{\mathrm{eq}}^{IT}$
10. *num*PQ = - $K_{\mathrm{m}}^{Q} * V_{\mathrm{r}}^{T} * K_{\mathrm{mB}}^{I} * V_{\mathrm{f}}^{A} * K_{\mathrm{i}}^{I} * [P]*[Q] / K_{\mathrm{eq}}^{AQ}$

11. *num*IB = $+ K_m^Q * V_r^T * K_{mB}^A * K_i^A * V_f^I * [I]*[B]$

12. *num*AB = $+ K_m^Q * V_r^T * K_{mB}^I * K_i^I * V_f^A * [A]*[B]$

Expressions obtained for denominator coefficients (*coef*)

1. *coef*BP^{2}T = $V_f^I * V_f^A * (K_m^Q)^2 * [B]*[P]^2*[T] /(V_r^Q * K_i\mathrm{B}^{IT} * K_{eq}^{AQ} * K_{eq}^{IT})$

2. *coef*BP^{2}Q = $V_f^I * V_r^Q * K_m^A * K_m^T * [B]*[P]^2*[Q] / (V_f^A * K_{iP}^{AQ} * K_{eq}^{IT})$

3. *coef*B^{2}PT = $K_m^I * (V_r^T)^2 * K_m^Q * K_m^A * [B]^2*[P]*[T]/ (K_{iP}^{IT} * K_m^T * V_f^I)$

4. *coef*B^{2}PQ = $K_m^I * V_r^T * K_m^A * V_r^Q * [B]^2*[P]*[Q]/ (K_{iP}^{AQ} * V_f^A)$

5. *coef*IBP^2 = $V_r^T * (K_m^Q)^2 * V_f^A * [I]*[B]*[P]^2 / (K_{eq}^{AQ} * K_{iP}^{IT} * V_r^Q)$

6. *coef*IB^{2}P = $K_m^A * V_r^T * K_m^Q * [I]*[B]^2*[P] / K_{iP}^{IT}$

7. *coef*ABP^2 = $K_m^Q * V_f^I * K_m^T * [A]*[B]*[P]^2 / (K_{eq}^{IT} * K_{iP}^{AQ})$

8. *coef*AB^{2}P = $K_m^I * V_r^T * K_m^Q * [A]*[B]^2*[P] / K_{iP}^{AQ}$

9. *coef*P^{2}T = $(K_m^Q)^2 * V_f^I * V_f^A * [P]^2*[T] /(K_{eq}^{AQ} * K_{eq}^{IT} * V_r^Q)$

10. *coef*P^{2}Q = $K_m^Q * V_f^A * V_f^I * K_m^T * [P]^2*[Q] /(K_{eq}^{AQ} * K_{eq}^{IT} * V_r^Q)$

11. *coef*BPT = $K_m^A * V_r^T * K_m^Q * [K_i^I * K_{mB}^I / (K_i^T * K_{mP}^T) + V_r^T * K_{mB}^A * K_i^A * K_m^I / (V_f^I * K_{iP}^{IT} * K_m^A * K_m^T) + V_f^A * K_m^Q * K_m^I / (K_{eq}^{AQ} * K_i^T * V_r^Q * K_m^A)] * [B][P][T]$

12. *coef*BPQ = $K_m^I * V_r^T * K_m^Q * [K_i^A * K_{mB}^A / (K_i^Q * K_{mP}^Q) + V_r^Q * K_{mB}^I * K_i^I * K_m^A / (V_f^A * K_m^I * K_m^Q * K_{iP}^{AQ}) + K_m^A * V_f^I * K_m^T / (K_{eq}^{IT} * K_i^Q * K_m^I * V_r^T)] * [B]*[P]*[Q]$

13. *coef*B^{2}T = $K_m^A * V_r^T * K_m^Q * K_m^I * [B]^2*[T] / K_i^T$

14. *coef*B^{2}Q = $K_m^I * V_r^T * K_m^Q * K_m^A * [B]^2*[Q] / K_i^Q$

15. *coef*IP^2 = $(K_m^Q)^2 * V_f^I * K_m^T * V_f^A * [I]*[P]^2 / (K_{eq}^{IT} * K_{eq}^{AQ} * V_r^Q * K_i^I)$

16. *coef*IBP = $K_m^A * V_r^T * K_m^Q * [V_f^I * K_m^T / (K_i^I * K_{eq}^{IT} * V_r^T) + K_{mB}^A * K_i^A / (K_m^A * K_{iP}^{IT}) + V_f^A * K_m^Q /(K_{eq}^{AQ} * K_m^A * V_r^Q)] * [I]*[B]*[P]$

17. *coef*IB^2 = $K_m^A * V_r^T * K_m^Q * [I]*[B]^2$

18. *coef*AP^2 = $(K_m^Q)^2 * V_f^A * V_f^I * K_m^T * [A]*[P]^2 / (K_{eq}^{AQ} * K_{eq}^{IT} * K_i^A * V_r^Q)$

19. *coef*ABP = $K_m^I * V_r^T * K_m^Q * [V_f^A * K_m^Q / (K_i^A * K_{eq}^{AQ} * V_r^Q) + V_f^I * K_m^T / (K_{eq}^{IT} * K_m^I * V_r^T) + K_{mB}^I * K_i^I / (K_m^I * K_{iP}^{AQ})] * [A]*[B]*[P]$

20. *coef*AB^2 = $K_m^I * V_r^T * K_m^Q * [A]*[B]^2$

21. *coef*PT = $V_r^T * K_m^Q [K_{mB}^A * K_i^A * V_f^I / (K_{eq}^{IT} * V_r^T) + K_{mB}^I * K_i^I * V_f^A * K_m^Q / (K_{eq}^{AQ} * K_i^T * V_r^Q)]* [P]*[T]$

22. *coef*PQ = $V_r^T * K_m^Q * [K_{mB}^I * K_i^I * V_f^A * / (K_{eq}^{AQ} * V_r^Q) + K_{mB}^A * K_i^A V_f^I * K_m^T / (K_{eq}^{IT} * K_i^Q * V_r^T)] * [P]*[Q]$

23. *coef*P^2 $= (K_m^Q)^2 * V_f^A * V_f^I * K_m^T [P]^2 / (K_{eq}^{IT} * K_{eq}^{AQ} * V_r^Q)$

24. *coef*BT $= K_m^A * V_r^T * K_m^Q * [K_{mB}^A * K_i^A * K_m^I / (K_i^T * K_m^A) + K_{mB}^I * K_i^I / K_i^T] * [B]*[T]$

25. *coef*BQ $= K_m^I * V_r^T * K_m^Q *[K_{mB}^I * K_i^I * K_m^A / (K_m^I * K_i^Q) + K_{mB}^A * K_i^A / K_i^Q] * [B]*[Q]$

26. *coef*BP $= K_m^I * V_r^T * K_m^Q *[V_f^A * K_m^Q / (K_{eq}^{AQ} * V_r^Q) + K_m^A * V_f^I * K_m^T / (K_{eq}^{IT} * K_m^I * V_r^T)] * [B]*[P]$

27. *coef*B^2 $= K_m^I * V_r^T * K_m^Q * K_m^A * [B]^2$

28. *coef*IP $= V_r^T * K_m^Q *[K_{mB}^A * K_i^A * V_f^I * K_m^T / (K_i^I * V_r^T * K_{eq}^{IT}) + K_{mB}^I * V_f^A * K_m^Q / (K_{eq}^{AQ} * V_r^Q)] * [I]*[P]$

29. *coef*IB $= V_r^T * K_m^Q * (K_{mB}^I * K_m^A + K_{mB}^A * K_i^A) * [I]*[B]$

30. *coef*AP $= V_r^T * K_m^Q * [K_{mB}^A * V_f^I * K_m^T / (K_{eq}^{IT} * V_r^T) + K_{mB}^I * K_i^I * V_f^A * K_m^Q / (K_i^A * K_{eq}^{AQ} * V_r^Q)] * [A]*[P]$

31. *coef*AB $= V_r^T * K_m^Q * (K_{mB}^A * K_m^I + K_{mB}^I * K_i^I) * [A]*[B]$

32. *coef*T $= V_r^T * K_m^Q * K_{mB}^I * K_{mB}^A * K_i^A * K_i^I * [T] / K_i^T$

33. *coef*Q $= V_r^T * K_m^Q * K_{mB}^A * K_{mB}^I * K_i^I * K_i^A * [Q] / K_i^Q$

34. *coef*P $= V_r^T * K_m^Q * [K_{mB}^A * K_i^A * K_m^T * V_f^I / (K_{eq}^{IT} * V_r^T) + K_{mB}^I * K_i^I * V_f^A * K_m^Q / (K_{eq}^{AQ} * V_r^Q)] * [P]$

35. *coef*B $= V_r^T * K_m^Q * (K_{mB}^A * K_i^A * K_m^I + K_{mB}^I * K_i^I * K_m^A) * [B]$

36. *coef*I $= V_r^T * K_m^Q * K_{mB}^I * K_{mB}^A * K_i^A * [I]$

37. *coef*A $= V_r^T * K_m^Q * K_{mB}^I * K_{mB}^A * K_i^I * [A]$

38. const $= V_r^T * K_m^Q * K_m B^I * K_m B^A * K_i^A * K_i^I$

The complete rate equation is shown in a condensed form in Additional File 1, Table S2.

Validation of XR rate equation

The obtained rate expression for CtXR (see Additional File 1, Table S2) can be reduced to the respective complete bi-substrate rate expressions (equivalent to Equation S1) describing NAD(H)- and NADP(H)-dependent interconversion of xylose and xylitol based on an ordered Bi Bi reaction mechanism (data not shown). After disregarding product formation the expression adopts as expected the form of the rate equation presented recently by [3].

Furthermore the equation was challenged by comparing calculated with experimentally obtained initial rates determined with CtXR under different conditions with respect to presence and concentrations of coenzymes. Initial concentrations of xylose were 133 mM while those of

coenzymes were varied as shown in Additional File 2, Figure S3. Measurements were carried out at 30°C in a 100 mM potassium phosphate buffer (PPB), pH 7.0. A purified fraction of CtXR (15 µmol/min/mg), prepared as described by [4], was used in all experiments. Results were presented together with a summary of initial reactant concentrations in Additional File 2, Fig. S3. Experimentally obtained initial rates correlated excellently with a slope of 1 and correlation coefficient of 0.95 with the corresponding calculated rates. Based on these results we concluded that the equation is correct and can be used to simulate enzyme kinetic behavior of CtXR.

Acquisition of model-relevant kinetic data

Kinetic parameters of CtXR were determined at 30°C in both directions of reaction from full kinetic studies carried out in 100 mM PPB, pH 7.0. Xylose (xylitol) and NAD(P)H ($NAD(P)^+$) were varied in a concentration range of 5 – 480 mM (20 – 1920 mM) and 6.3 – 200 µM (NAD^+: 0.06 – 2.00 mM; $NADP^+$: 6.3 – 200 µM), respectively. Values for kinetic constants V_{max} (maximal rate), K_{iA} (dissociation constant representing coenzyme binding to the free enzyme) and Michaelis constants for coenzyme (K_{mA}) and substrate (K_{mB}) were obtained by non-linear fits of experimental data with Equation S1 representing the rate equation for an ordered Bi Bi mechanism.

$$v = V_{max}\,[A][B]/(K_{iA}K_{mB} + K_{mA}[B] + K_{mB}[A] + [A][B]) \qquad \text{(S1)}$$

Because K_{iA}'s for $NADP^+$ and NADPH obtained from Equation S1 were very low (1-5 µM) and afflicted with high standard deviation we determined representative K_{iA}'s by fluorescence spectroscopy following a reported protocol [5]. Validity of resultant values was tested by comparing the K_{eq} calculated by the Haldane relationship from respective kinetic parameters with that obtained from experiments (see Table 2). Experimental K_{eq}'s were determined for R_1 and R_2 at 30°C in 100 mM PPB, pH 7.0 in accordance to a reported protocol [6]. Initial concentrations of xylose, xylitol, $NAD(P)^+$ and NAD(P)H were 100 mM, 1000 mM, 85 µM and 92 µM, respectively and 0.2 mg/mL of purified CtXR (15 µmol/min/mg) were applied. Dissociation constants for xylitol (K_{iP}) were determined at constant saturating concentrations of NAD(P)H (= 0.2 mM) and varying concentrations of xylose (5 - 470 mM) and xylitol (0 – 2000 mM). Values for K_{iP}'s were obtained from non-linear fits of Equation S2 to respective experimental data.

$$v = V_{max}\,[B]/\{K_{mB}\,(1 + K_{iA}/[A])(1 + K_{mQ}[P]/(K_{iQ}K_{mP})) + [B]\,(1 + K_{mA}/[A] + [P]/K_{iP})\} \qquad \text{(S2)}$$

Values for K_{iA}, K_{mA}, K_{mQ}, K_{iQ} and K_{mP} as well as concentrations for [NAD(P)H] (= 0.2 mM) were constrained to that obtained from full kinetic studies (see Table 2). Values for K_{iB}'s representing the dissociation constants of xylose were calculated from respective Haldane relationships.

Michaelis constants for xylitol and NAD^+ were determined from cell-free extracts of BP000 and *C. tenuis*. $K_{m,xylitol}$ (K_{m,NAD^+}) was measured at constant and saturating concentrations of NAD^+(xylitol) (3 mM, (250 mM)) while concentrations of xylitol (NAD^+) were varied between 5 – 250 mM (0.03 – 3.00 mM). As resultant values for $K_{m,xylitol}$ of 16 ± 2 mM (BP000) and 15 ± 2 (*C. tenuis*) and for K_{m,NAD^+} of 0.12 ± 0.03 mM (BP000) and of 0.2 ± 0.05 mM (*C. tenuis*) were very similar the set of kinetic parameters reported for GmXDH was applied for both strains (see Table 2) [7, 8].

Michaelis constants of $NADP^+$ (K_{NADP^+}) and competitive inhibition by NADPH ($K_{i,NADPH}$) were analyzed for reactions catalyzed by ZWF1 and GND from cell-free extracts of BP000 and *C. tenuis*. Concentrations of $NADP^+$ (0.05 – 3 mM) and NADPH (0 – 0.2 mM) were varied while those of glucose 6-phosphate (18.0 mM) and 6-phosphogluconate (1.0 mM) were constant and saturating. Values for K_{NADP^+} and $K_{i,NADPH}$ were obtained from fitting experimental data by Equation S3 describing linear competitive inhibition.

$$v=[NADP^+]V_{max}/\{K_{NADP^+}(1+[NADPH]/K_{i,NADPH}) + [NADP^+]\} \qquad \text{(S3)}$$

Nonlinear regressions were carried out with SigmaPlot 9.0.

Activity of CtXR with DHAP

Kinetic parameters were determined from initial rates recorded as decrease in NADH absorption at 25°C. Reactions were carried out in 50 mM PPB pH 7.0. Concentrations of DHAP were varied (1.37 – 8.00 mM) while initial concentrations of NADH were 250 µM. CtXR purified to apparent homogeneity was used.

CtXR could not be saturated by DHAP and initial rates displayed a linear dependency on the DHAP concentration within the concentration range addressed. A catalytic efficiency of 74 $M^{-1}s^{-1}$ was obtained. For comparison a catalytic efficiency of 137 $M^{-1}s^{-1}$ was reported for CtXR when xylose was reduced [5].

References

1. Klimacek M, Krahulec S, Sauer U, Nidetzky B. Limitations in xylose-fermenting *Saccharomyces cerevisiae*, made evident through comprehensive metabolite profiling and thermodynamic analysis. Applied and environmental microbiology. 2010;76(22):7566-74. doi:10.1128/AEM.01787-10.
2. Segel IH. Enzyme kinetics. Behavior and analysis of rapid equilibrium and steady-state enzyme systems. New York: John Wiley and Sons, Inc.; 1993.
3. Banta S, Boston M, Jarnagin A, Anderson S. Mathematical modeling of in vitro enzymatic production of 2-Keto-L-gulonic acid using NAD(H) or NADP(H) as cofactors. Metab Eng. 2002;4(4):273-84.
4. Neuhauser W, Haltrich D, Kulbe KD, Nidetzky B. NAD(P)H-dependent aldose reductase from the xylose-assimilating yeast Candida tenuis. Isolation, characterization and biochemical properties of the enzyme. The Biochemical journal. 1997;326 (Pt 3):683-92.
5. Nidetzky B, Klimacek M, Mayr P. Transient-state and steady-state kinetic studies of the mechanism of NADH-dependent aldehyde reduction catalyzed by xylose reductase from the yeast *Candida tenuis*. Biochemistry. 2001;40(34):10371-81.
6. Bubner P, Klimacek M, Nidetzky B. Structure-guided engineering of the coenzyme specificity of *Pseudomonas fluorescens* mannitol 2-dehydrogenase to enable efficient utilization of NAD(H) and NADP(H). FEBS letters. 2008;582(2):233-7. doi:10.1016/j.febslet.2007.12.008.
7. Lunzer R, Mamnun Y, Haltrich D, Kulbe KD, Nidetzky B. Structural and functional properties of a yeast xylitol dehydrogenase, a Zn2+-containing metalloenzyme similar to medium-chain sorbitol dehydrogenases. The Biochemical journal. 1998;336 (Pt 1):91-9.
8. Nidetzky B, Helmer H, Klimacek M, Lunzer R, Mayer G. Characterization of recombinant xylitol dehydrogenase from *Galactocandida mastotermitis* expressed in *Escherichia coli*. Chemico-biological interactions. 2003;143-144:533-42.
